# Complications after surgical management of proximal humeral fractures: a systematic review of event terms and definitions

**DOI:** 10.1186/s12891-020-03353-8

**Published:** 2020-05-26

**Authors:** N. Alispahic, S. Brorson, C. Bahrs, A. Joeris, A. Steinitz, L. Audigé

**Affiliations:** 1 Department of Orthopedic Surgery and Traumatology, University Hospital of Basel, Basel, Switzerland; 2 Department of Orthopedic Surgery, Zealand University Hospital and Department of Clinical Medicine, University of Copenhagen, Copenhagen, Denmark; 3 Department of Traumatology and Reconstructive Surgery, Eberhard Karls University Tübingen, BG Trauma Center Tübingen, Tübingen, Germany; 4 AO Clinical Investigation and Documentation, Dübendorf, Switzerland; 5 Crossklinik, Basel, Switzerland; 6 Research and Development Department, Shoulder and Elbow Surgery, Schulthess Clinic, Zurich, Switzerland; 7 Institute for Clinical Epidemiology and Biostatistics, University Hospital of Basel, Basel, Switzerland

**Keywords:** Proximal humeral fractures, Surgical treatment, Unfavorable events, Adverse events, Complications, Systematic literature review, Standardization

## Abstract

**Background:**

The most frequently used surgical procedures for treating a proximal humeral fracture (PHF) are plate osteosynthesis, nail osteosynthesis and arthroplasty. Evidence-based recommendations for an appropriate surgical procedure after PHF requires transparent and valid safety data. We performed a systematic review to examine reported terms and definitions of complications after surgically-treated PHFs.

**Methods:**

A literature search was conducted on PubMed, Cochrane Library, EMBASE, Scopus and WorldCat to identify clinical articles and book chapters on complications of PHF published from 2010 to 2017. Complication terms and definitions were extracted from each selected article independently by two reviewers and grouped according to a predefined scheme.

**Results:**

From 1376 initial references, we selected 470 articles, of which 103 were reviewed in reverse chronological order until no further information was gained. Twelve book chapters were reviewed. We found 667 local event terms associated with complications after surgical treatment of PHFs. The most frequently used event terms were infection (52 references), nonunion (*n* = 42), malunion (*n* = 35), avascular necrosis (*n* = 27) and pain (*n* = 25). Overall, 345, 177, 257 and 102 local event terms were related to plating, nailing, arthroplasty and other surgical techniques, respectively. Radiological assessment was the basis for the majority of event terms and complication definitions. Thirty-six event definitions were extracted, mostly defining the terms “secondary fracture displacement”, “screw perforation/cutout”, “malunion”, “delayed healing” and “notching”.

**Conclusion:**

Scientific literature on surgically-managed PHF uses different terms to describe complications and without approved definitions, which highlights a lack of agreement on adverse event terminology for PHFs. Defined event terms are mostly based on radiological observations. Consensus among shoulder surgeons on a core event set is indispensable to support the standardization of safety reporting for surgically-treated PHFs.

## Background

Proximal humeral fractures (PHFs) account for 4 to 6% of all fractures [1]. The majority of cases are seen in older patients and associated with osteoporosis [2]. While the management of nondisplaced fractures involves nonoperative procedures, displaced fractures can be treated surgically by plate osteosynthesis, intramedullary nail fixation or arthroplasty using a wide variety of prostheses. Standardized outcome reporting, particularly of safety events or complications, is necessary in order to compare the different surgical procedures [3, 4] and foster evidence-based decision making in orthopedic surgery [5, 6].

In orthopedic surgery, approaches for the standardization of complication definitions have been proposed for several indications including distal radius fractures [7], knee arthroplasty [8], spine surgery [9], arthroscopic rotator cuff repair [10] and shoulder arthroplasty [11, 12]. While complication reporting is essential to evaluate the quality of health care [13], current guidelines provide neither support for reporting complication events nor consensus definitions for these events [3]. A common understanding of complications would be very important in PHF management, notably to assess causal factors. For example, a rotator cuff tear sustained after intramedullary nail treatment of a PHF may be considered either as surgery-related because of an iatrogenic lesion caused by the implant or disease-related due to the aging and degenerative processes of an older patient.

To support the standardization of complication reporting in PHF treatment, we conducted a systematic literature review of event terms and definitions of complications after these fractures. While events associated with nonoperative management have been previously discussed [14], this report focuses on events reported in the context of surgical treatment options for PHFs.

## Methods

A systematic literature search of peer-reviewed articles and book chapters focusing on adverse events and complications after PHF treatment was implemented and reported according to the PRISMA guidelines [15]. Only published clinical research in humans and reviews of these studies were included. In June 2017, we searched the PubMed, Cochrane Library, EMBASE and Scopus databases for scientific articles published after 2009. Book chapters were identified in a WorldCat online library search limited to the period from 2016 to 2017. Publications in English, German or French were included.

A qualified librarian generated the search algorithm for each database and provided the initial reference list (Supplementary File 1). Two reviewers (first and fifth authors) made a preliminary selection based on the reference titles and abstracts. A third reviewer (senior author) examined all ambiguous references to make a final decision on whether they could be included in the review or not. Full-text articles were then retrieved and reviewed in reverse chronological order starting from 2017, while considering successive batches of 20 randomly selected papers during data extraction. We stopped collecting data when all three reviewers reached a consensus that further extraction would very likely provide no new information for the project. A similar approach was used for the selected book chapters.

Throughout this work, we use either the term “unfavorable event” or “event” to describe both an “adverse event” or “complication” without attempting to make a distinction between them. We extracted qualitative data comprising all mentioned event terms and reported definitions of single events. Each full-text article or book chapter was reviewed by one of the authors; extracted data was double-checked by another author, and the senior author addressed any ambiguous terms. Event terms were classified according to the following treatment modalities of plate osteosynthesis, intramedullary nail fixation, arthroplasty, other surgical procedures and nonoperative management, the latter of which is already presented [14] and thus excluded from further analyses. References that were quoted in relation to a specific definition were also reviewed to assess the exactness of the citations.

Data were managed in REDCap [16] (Version 6.16.5,© 2018 Vanderbilt University, USA) and exported into Stata Intercooled (Version 14, Stata Corp SA, Texas, USA) for descriptive analyses and event listing. For this report, we focused on all events that were extracted and defined as exclusively related to operative management; the event terms were listed and organized according to preassigned event groups adapted from existing consensus on rotator cuff repair [10] and shoulder arthroplasty [11]. In short, events were distinguished between those considered as local / regional to the injured shoulder and non-local (i.e. events affecting any part of the rest of the body). Local events were further categorized into one of nine event groups including: implant/device events, osteochondral events, shoulder instability, pain, surgical site infection, peripheral neurologic events, vascular events, superficial soft tissue events, and deep soft tissue events including rotator cuff problems. Non-local events were categorized according to the organ system they directly affect, but not considered further in this report. For each event term, the number of citing references was reported to indicate which terms may represent a more common language.

## Results

**Fig. 1:**
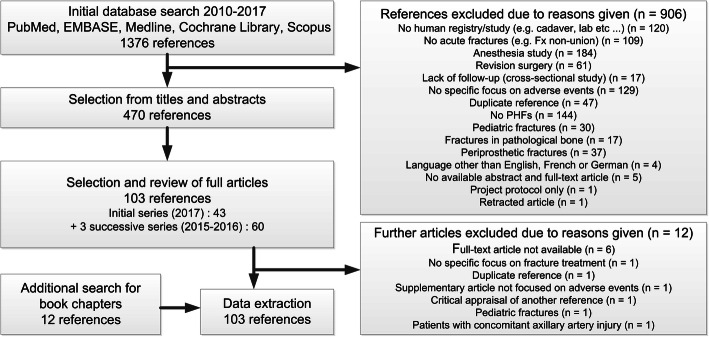
Systematic review article selection flowchart. PHF = proximal humeral fracture. Reprinted with permission from Brorson et al. [14]

From 1376 initial references, we collated 470 articles for full-text review after excluding 906 references (Fig. 1). For the data extraction process, a further 12 articles were excluded, which gave rise to a total of 103 articles including 12 book chapters for assessment (Supplementary File 2).

We extracted 694 local event terms. After a second review, 39 terms were excluded from our initial list because they were considered either as treatment options (e.g. “need for secondary surgery”, “hardware removal”), outcomes of events (e.g. “loss of motion”, “death”) or too general in their description (e.g. “implant-related problems”, “perioperative morbidity”) (Supplementary File 3). Our final list of local event terms comprised a total of 457 terms with the majority listed under the groups of local implant events and osteochondral events (Table 1). The 10 most commonly reported event terms extracted from between 16 and 56 publications were: “dislocation”, “implant failure”, “impingement”, “loss of reduction”, “stiffness”, “pain”, “avascular necrosis”, “malunion” and “infection”. Based on the various surgical treatment options, there were 345, 177, 257 and 102 event terms reported in the context of plating, nailing, arthroplasty and other surgical techniques (mostly K-wire fracture fixation), respectively. While some event terms were mentioned for all procedures (e.g. “implant failure”, “nonunion” or “infections”), other terms were more specific to a surgical method; “luxation” and “acromion fracture” were associated with shoulder arthroplasty in 4 and 9 references respectively, whereas “notching”—an event that is pathognomonic of reverse shoulder arthroplasty—was extracted from 27 references. Multiple terms were used to describe single events, for example, there were 12 different event terms used to describe “notching” in 25 articles and book chapters. Only a small number of events such as “screw perforation/penetration” were categorized as either an intra- (38 event terms from 75 articles) or postoperative event (16 event terms from 31 articles).

**Table 1:** Number of reported event terms per event group and specification according to treatment options with the related number of references

Event groups^a^	Specifications	Number of extracted terms					Number of references
		P	N	A	OS	All	
1- Implant events	Migration (subsidence, tilt, shift)	2	2	8	8	20	18
	Radiolucency around the implant / implant loosening	7	3	18	3	26	27
	Breakage	17	4	1	2	20	37
	Disassembly	1	–	–	–	1	1
	Malpositioning	7	7	10	1	21	15
	Screw or bolt back-out / loosening	16	10	–	2	24	22
	Hardware local irritation	2	–	–	–	2	2
	Other specific terms	2	1	2	1	3	3
2- Osteochondral events	Bone formation / resorption	2	2	19	2	19	27
	Arthritis	8	3	3	2	14	13
	Fracture around the implant	5	7	25	–	35	28
	Screw / bolt perforation / cutout	46	18	2	4	58	64
	Tuberosity migration / resorption	8	5	29	3	39	37
	Osteonecrosis	20	13	3	8	26	78
	Delayed / nonunion / malunion	27	18	27	11	52	83
	Malreduction / loss of reduction / fracture displacement	65	23	14	17	97	75
3- Shoulder instability	General terms	1	1	4	–	6	13
	Subluxation	–	–	1	–	1	2
	Dislocation	2	2	7	1	9	24
4- Shoulder pain (idiopathic)	General terms	5	3	3	1	7	33
5- Peripheral neurological events	General terms	3	2	5	–	6	10
	Sensory and/or motor disturbance: cervical or brachial plexus	2	1	4	1	5	5
	Sensory and/or motor disturbance: branch neuropathy	27	7	15	13	42	41
	Autonomic disturbance: CRPS	2	3	6	1	7	12
6- Vascular events	Hematoma which requires evacuation by needle or surgery	2	1	4	1	4	20
	Superficial and deep thrombosis at the involved extremity	1	1	2	2	3	3
	Other specific terms	4	–	4	2	10	10
7- Infections	Not specified	3	2	3	2	4	55
	SSI: superficial	7	3	4	1	9	19
	SSI: deep	6	2	3	5	11	26
	Late hematogenous infections	–	–	1	–	1	1
8- Superficial soft tissue events	Early events	5	1	1	–	7	6
	Late events: hypertrophic scar and keloid	2	1	–	2	4	3
9- Deep soft tissue events	Affecting the subacromial / subcoracoidal space	17	5	4	2	23	49
	Affecting the biceps	3	2	1	1	5	4
	Affecting the capsule (shoulder stiffness, metallosis)	8	5	4	4	11	38
	Affecting the rotator cuff	9	19	15	1	34	34
	Affecting the deltoid	1	–	2	–	3	3
	Other terms	–	–	2	–	2	2

*P* plate osteosynthesis, *N* intramedullary nail fixation, *A* arthroplasty, *OS* other surgical techniques, *All* all surgical treatment options combined, *CRPS* complex regional pain syndrome, *SSI* surgical site infections

^a^Event groups were considered from a previous international consensus process for arthroscopic rotator cuff repair [10] and shoulder arthroplasty [11]

We did not find a general complication definition inherent in the surgical treatment of PHFs. Thirty-six definitions of single events were identified (Supplementary File 4). Most of these definitions were based on radiographic parameters such as 13 different definitions of “secondary fracture displacement” related to displacement of the tuberosities or the humeral head or the humeral neck in varus. The term “stiffness” (e.g. limitation of active and passive motion compared to contralateral shoulder in at least 2 directions including forward flexion < 120° or 50% restriction of contralateral external rotation and internal rotation or a passive anterior elevation < 80°) was defined solely based on clinical assessments.

## Discussion

The aim of this review was to analyze articles on the surgical management of PHF according to reported terms and definitions of adverse events or complications, which we jointly termed “unfavorable events”. None of the reviewed articles presented a general definition of the term “complication”. We identified and categorized 694 different event terms and listed only 36 term definitions. This shows a lack of consensus in the use of terms and definitions of unfavorable events associated with PHF surgical treatment. This is a challenge for policy makers requiring valid safety assessments of surgical interventions for PHF, and for both patients and clinicians aiming at a well-founded, decision-making process in treating these fractures.

In order to arrange the event terms, we used a classification system that was recently developed by consensus in shoulder surgery with a high level of agreement [11, 17]. All event terms could be categorized, which provided a good overview of the various events mentioned in the clinical PHF literature. The process of classifying the terms into local and non-local events was straightforward, especially when local events such as nonunion, avascular necrosis, loss of reduction or implant failure were defined based on radiographic assessments. Further differentiation of local events into event groups was sometimes challenging, and our suggestion should serve as a basis to develop an international consensus. For instance, when considering implant and osteochondral events, we allocated the terms “cut-out” and “screw perforation/penetration” to the osteochondral event group as they were considered an injury to the osteochondral tissue instead of a direct failure of the implant. On the other hand, the event term “screw back-out” is an implant event because it causes direct instability of the osteosynthesis and only indirectly of the fracture itself. In addition, we chose to review all event terms together, while simultaneously indicating in which treatment context they were mentioned. This allows the classifier to recognize which events would relate to all or one specific surgical type (e.g. internal fixation versus arthroplasty) or the use of a specific implant (e.g. nail or plate). This can be well illustrated with the event terms “cutout” and “humeral head necrosis”. “Cutout” was extracted from 31 references of which 21 were attributed to plate osteosynthesis alone, one to nail osteosynthesis alone and nine for both plate and nail osteosynthesis. “Humeral head osteonecrosis” was mentioned in 43 references focused on osteosynthesis, 17 of which addressed plate osteosynthesis alone, three nail osteosynthesis alone and 23 both procedures.

In this review, unfavorable events were only assessed based on their descriptive terms without inferring on their treatment modality and consequences for the patient. In particular, specific radiographic patterns may be used to define unfavorable events or complications, but do not necessarily impact on the functional outcome and expectations reported by the patient. For example, PHF-associated humeral head necrosis is well tolerated by many patients. In this sense, humeral head necrosis is not necessarily an adverse event from the patient’s perspective. Another example is that of secondary fracture displacement defined by standardized radiography diagnostics; this event may not be of primary concern for older patients with limited functional demands. More knowledge is needed to clarify the association between patient-reported outcomes and radiographically-defined complications after the surgical management of PHFs.

Some unfavorable events such as “malreduction” or “screw perforation” were defined in terms that do not presume the timing of occurrence, although they may take place either during or after surgery. Such attributes of timing (i.e. the designation of a fixed time point(s)) is relevant and has been defined for any event by consensus based on an existing definition in surgery [17]. Our distinction regarding fracture reduction, for instance, was that the “primary reduction problem” occurred intraoperatively and the “secondary reduction problem” occurred postoperatively. Some extracted definitions, however, clearly refer to the postoperative period; one such definition is “varus collapse” defined as “a change of the head-shaft angle of less than 120° from the first postoperative x-ray to the final follow up” [18, 19].

While the quality of systematic literature reviews always depends on the quality of the included studies, we examined many different studies ranging in evidence from Level I to IV. The inclusion of all clinical article types was necessary to retrieve the current terminology and definitions. In our review, Level I studies cannot be considered superior over case series, since this work was qualitative and did not aim at quantifying complication rates in the surgical treatment of PHF. The number of reviewed articles may be considered limited and reflective of only a small proportion of the published PHF literature. However, based on previous experience [20, 21], our strategy of focusing on the most recent publications to source the most relevant and common event terms and definitions can be considered sufficient and most effective. The review of textbooks was restricted to a limited time period for the same reason. Checking quoted references for the evaluation of retrieved definitions was essential to assess whether reported definitions were original or modified. Also, the retrieval of event terms could be influenced by the reviewer’s judgment, particularly if any term described an unfavorable event. Nonetheless, all extracted terms were assessed based on a proposal to ensure the consistency of extraction between reviewers [3]; our final list was cross-checked and agreed by consensus between reviewers. Finally, the applied classification of subgroups based on clinical presentation was not a straightforward process because different terms can be categorized into different subgroups.

## Conclusion

Scientific literature on the surgical treatment of PHFs report a wide variety of terms to describe unfavorable events (adverse events / complications) without approved definitions, which highlights a lack of agreement on adverse event terminology for PHFs. Defined event terms are mostly based on radiological observations. Consensus on a core event set, which is held among shoulder surgeons and considers the involvement of patient representatives, is indispensable to support the standardization of safety reporting for surgically-treated PHFs.

## Supplementary information

**Additional file 1.** Search protocol for proximal humerus fractures in EMBASE, Medline, PubMed, Scopus and Cochrane databases.

**Additional file 2.** List of reviewed scientific articles and book chapters.

**Additional file 3.** Extracted unfavorable event terms.

**Additional file 4.** Extracted definitions related to specific event terms.

## Data Availability

The datasets during and/or analyzed during the current study are available from the corresponding author on reasonable request.

## Abbreviation

PHF: Proximal humerus fracture

## References

[1] Court-Brown CM, Garg A, McQueen MM (2001). The epidemiology of proximal humeral fractures. Acta Orthop Scand.

[2] Olsson C, Nordqvist A, Petersson CJ (2004). Increased fragility in patients with fracture of the proximal humerus: a case control study. Bone.

[3] Audige L, Goldhahn S, Daigl M, Goldhahn J, Blauth M, Hanson B (2014). How to document and report orthopedic complications in clinical studies? A proposal for standardization. Arch Orthop Trauma Surg.

[4] Gargon E, Gurung B, Medley N, Altman DG, Blazeby JM, Clarke M, Williamson PR (2014). Choosing important health outcomes for comparative effectiveness research: a systematic review. PLoS One.

[5] Spindler KP, Kuhn JE, Dunn W, Matthews CE, Harrell FE, Dittus RS (2005). Reading and reviewing the orthopaedic literature: a systematic, evidence-based medicine approach. J Am Acad Orthop Surg.

[6] Suk M, Norvell DC, Hanson B, Dettori JR, Helfet D (2008). Evidence-based orthopaedic surgery: what is evidence without the outcomes?. J Am Acad Orthop Surg.

[7] McKay SD, MacDermid JC, Roth JH, Richards RS (2001). Assessment of complications of distal radius fractures and development of a complication checklist. J Hand Surg Am.

[8] Healy WL, Della Valle CJ, Iorio R, Berend KR, Cushner FD, Dalury DF, Lonner JH (2013). Complications of total knee arthroplasty: standardized list and definitions of the knee society. Clin Orthop Rel Res.

[9] Mirza SK, Deyo RA, Heagerty PJ, Turner JA, Lee LA, Goodkin R (2006). Towards standardized measurement of adverse events in spine surgery: conceptual model and pilot evaluation. BMC Musculoskelet Disord.

[10] Audige L, Flury M, Muller AM, Panel ACC, Durchholz H (2016). Complications associated with arthroscopic rotator cuff tear repair: definition of a core event set by Delphi consensus process. J Shoulder Elb Surg.

[11] Audigé Laurent, Schwyzer Hans-Kaspar, Durchholz Holger, Äärimaa Ville, Alta Tjarco D., Amaral Marcus Vinicius, Armstrong Alison, van Noort Arthur, Bale Steve, Beyth Shaul, Bischof Andreas, Bokor Desmond J., Borroni Mario, Brorson Stig, Brownson Peter, Buchmann Stefan, Buess Eduard, Cass Benjamin, Kelly Cormac, De Cupis Vincenzo, Debeer Philippe, van Deurzen Derek F.P., Dillon Mark T., Durchholz Holger, Ekelund Anders, Etzner Mikael, Flury Matthias, Frankle Mark, Geoghegan John, Georgousis Harry, Gerber-Popp Ariane, Gulyás Károly, Henry Patrick, Hertel Ralph, Heuberer Philipp, Holland Philip, Holzer Nicolas, Hoy Greg, Imhoff Andreas B., Johannsen Hans Viggo, Kent Matthew, Kohut Georges, Lädermann Alexandre, Lambert Simon, Lanz Ulrich, Lederman Evan, Lehmann Lars, Leuzinger Jan, Lichtenberg Sven, Livesey Jonathan, Loew Markus, Lorbach Olaf, Lundgreen Kirsten, Maier Dirk, Martetschläger Frank, Matis Nicholas, Mehta Saurabh Sagar, Meyer Dominik, Millett Peter J., Moroder Philipp, Motta Geraldo, Mueller Andreas, Navarro Ronald A., Nebelung Wolfgang, Neumann Jörg, Page Richard, Paladini Paolo, Patel Vipul, Penning Ludo, Petré Dirk, Petriccioli Dario, Huijsmans Pol, Rangan Amar, Rees Jonny, Reinares Felipe, Resch Herbert, Romeo Anthony A., Rosso Claudio, Rotini Roberto, Ruiz-Iban Miguel A., Salomonsson Björn, Sandow Michael, Savoie Felix H., Scheer Johan, Scheibel Markus, Schwyzer Hans-Kaspar, Soza Rex Jose Francisco, Sperling John, Spormann Christoph, Tauber Mark, Thillemann Theis, Throckmorton Thomas, Peckham Tim, Toro Felipe, van der Pluijm Marco, van der Zwaal Peer, Visser Cornelis, Wambacher Markus, Weber Stephen C., Williams Gerald (2019). Core set of unfavorable events of shoulder arthroplasty: an international Delphi consensus process. Journal of Shoulder and Elbow Surgery.

[12] Durchholz H, Salomonsson B, Moroder P, Lambert S, Page R, Audigé L, on behalf of the SA Monitoring Steering Group (2019). Core set of radiological parameters for shoulder arthroplasty monitoring: criteria defined by an international Delphi consensus process. JBJS Open Access.

[13] Veen EJ, Janssen-Heijnen ML, Bosma E, de Jongh MA, Roukema JA (2012). The accuracy of complications documented in a prospective complication registry. J Surg Res.

[14] Brorson S, Alispahic N, Bahrs C, Joeris A, Steinitz A, Audige L (2019). Complications after non-surgical management of proximal humeral fractures: a systematic review of terms and definitions. BMC Musculoskelet Disord.

[15] Foster RL (2012). Reporting guidelines: CONSORT, PRISMA, and SQUIRE. J Spec Pediatr Nurs.

[16] Harris PA, Taylor R, Thielke R, Payne J, Gonzalez N, Conde JG (2009). Research electronic data capture (REDCap)--a metadata-driven methodology and workflow process for providing translational research informatics support. J Biomed Inform.

[17] Audige L, Flury M, Muller AM, Durchholz H, ARCR CES Consensus Panel (2016). Complications associated with arthroscopic rotator cuff tear repair: definition of a core event set by Delphi consensus process. J Shoulder Elb Surg.

[18] Brunner F, Sommer C, Bahrs C, Heuwinkel R, Hafner C, Rillmann P, Kohut G, Ekelund A, Muller M, Audige L (2009). Open reduction and internal fixation of proximal humerus fractures using a proximal humeral locked plate: a prospective multicenter analysis. J Orthop Trauma.

[19] Sohn HS, Jeon YS, Lee J, Shin SJ (2017). Clinical comparison between open plating and minimally invasive plate osteosynthesis for displaced proximal humeral fractures: a prospective randomized controlled trial. Injury.

[20] Audige L, Blum R, Muller AM, Flury M, Durchholz H (2015). Complications following arthroscopic rotator cuff tear repair: A systematic review of terms and definitions with focus on shoulder stiffness. Orthop J Sports Med.

[21] Jacxsens M, Walz T, Durchholz H, Muller AM, Flury M, Schwyzer HK, Audige L (2017). Towards standardised definitions of shoulder arthroplasty complications: a systematic review of terms and definitions. Arch Orthop Trauma Surg.

