# Supplementary material for: Complications after surgical management of proximal humeral fractures: a systematic review of event terms and definitions
Source: BMC Musculoskelet Disord. 2020 May 26;21:327. doi: 10.1186/s12891-020-03353-8 (PMC7251821; doi:10.1186/s12891-020-03353-8)
Supplement: Supplementary file 1 — Additional file 1. Search protocol for proximal humerus fractures in EMBASE, Medline, PubMed, Scopus and Cochrane databases. [file 12891_2020_3353_MOESM1_ESM.pdf]

Search Results for *Proximal Humerus Fractures*
Search protocols:

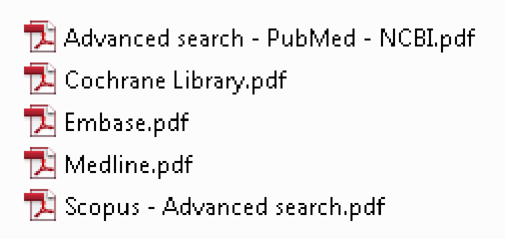

	**References**	**References after Deduplication**
**EMBASE**	1350	459
**Medline**	1302	1241
**PubMed**	163	68
**Scopus**	1576	295
**Cochrane**	75	17
**Pool**	4466	2080

Reference files: Proximal_humerus_fractures.enlx

before deduplication:

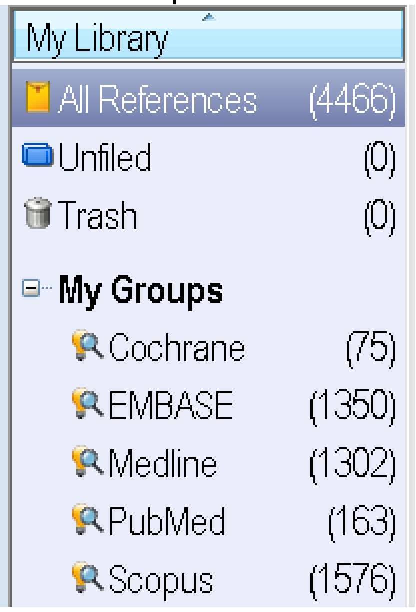

after:

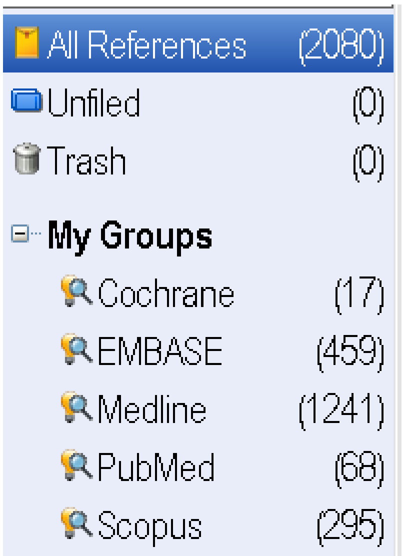

Don't miss the next webinar! Learn how drug analytics using Embase can inform drug development, repositioning and safety on July 27 at 4:00 pm CET Register now

Embase®

Register Login

Search Mapping Date Sources Fields Quick limits EBM Pub. types Languages Gender Age Animal Search tips

Results Filters

+ Expand — Collapse all Apply

- Sources
- Drugs
- Diseases
- Devices
- Floating Subheadings
- Age
- Gender
- Study types
- Publication types
- Journal titles
- Publication years
- Authors
- Conference Abstracts
- Drug Trade Names
- Drug Manufacturers
- Device Trade Names
- Device Manufacturers

Apply

History Save | Delete | Print view | Export | Email

Combine using And Or Collapse

#20	#3 AND #16 NOT [conference abstract]/lim AND ([english]/lim OR [french]/lim OR [german]/lim)	1,350
#19	#3 AND #16 NOT [conference abstract]/lim	1,483
#18	#3 AND #16 AND [conference abstract]/lim	96
#17	#3 AND #16	1,579
#16	#4 OR #15	5,072,247
#15	**'hematoma'**/exp OR **'infection rate'**/exp OR **'frozen shoulder'**/exp OR **'shoulder impingement syndrome'**/exp OR **'rotator cuff injury'**/exp OR **'neurological complication'**/exp OR **'femur head necrosis'**/exp OR **'screw loosening'**/exp OR **'reoperation'**/exp OR **'operative blood loss'**/exp OR **'postoperative hemorrhage'**/exp OR **'blood transfusion'**/exp OR **'pneumothorax'**/exp OR **infection**:ab,ti OR **impingement**:ab,ti OR **'frozen shoulder'**:ab,ti OR **breakage**:ab,ti OR **necrosis**:ab,ti OR ((**screw** OR **wire**) NEAR/3 (**perforation** OR **cutout** OR **'cut out'** OR **loosening** OR **backing**)):ab,ti OR (**'rotator cuff'** NEAR/3 (**lesion** OR **injury** OR **tendinopath*** OR **disease** OR **disorder*** OR **symptom*** OR **tear**)):ab,ti OR ((**implant** OR **prosthes*** OR **plate**) NEAR/5 (**loosen*** OR **tear*** OR **wear** OR **migration** OR **subluxation** OR **luxation** OR **removal** OR **displace*** OR **fail*** OR **dislocat*** OR **instab*** OR **malpositioning** OR **loosening**)):ab,ti OR ((**wound** OR **implant** OR **prosthetic**) NEAR/5 **infect***):ab,ti OR (**septic** NEAR/5 (**arthritis** OR **prosthes***)):ab,ti OR **osteolysis**:ab,ti OR **impinge***:ab,ti OR **stiffness**:ab,ti OR **hematoma***:ab,ti OR **haematoma***:ab,ti OR **thrombos***:ab,ti OR **impair***:ab,ti OR **palsy**:ab,ti OR **palsies**:ab,ti OR **disabilit***:ab,ti OR **mortality**:ab,ti OR **death**:ab,ti OR **dead**:ab,ti OR **reoperation***:ab,ti OR **pneumothorax**:ab,ti OR **refracture***:ab,ti OR **malunion**:ab,ti OR **'mal union'**:ab,ti OR **nonunion**:ab,ti OR **'non union'**:ab,ti OR **'fracture displacement'**:ab,ti OR **'scapular notching'**:ab,ti OR **'heterotopic ossification'**:ab,ti OR **'post-traumatic osteoarthritis'**:ab,ti OR **'posttraumatic osteoarthritis'**:ab,ti OR (**blood** NEAR/3 (**loss** OR **transfusion**)):ab,ti OR (**fixation** NEAR/3 (**problem*** OR **loss**)):ab,ti OR (**tuberosity*** NEAR/3 (**resorption** OR **dislocation** OR **migration** OR **reduction** OR **displacement**)):ab,ti OR **'glenoid erosion'**:ab,ti OR (**gleno*** NEAR/3 **degenerat***):ab,ti	3,897,460
#4	**'adverse outcome'**/exp OR **'treatment failure'**/exp OR **'postoperati** [illegible] OR **complication***:ab,ti OR (**adverse** NEAR/3 (**effect*** OR **event*** OR **outcome** OR **reaction***)):ab,ti OR (**postoperative** NEAR/3 (**complication*** OR **pain** OR **fail*** OR **injur*** OR **fractur***)):ab,ti OR ((**persistent** OR **movement** OR **shoulder** OR **syndrome**) NEAR/3 **pain**):ab,ti	
#3	#1 AND #2	2,202
#2	**'shoulder arthroplasty'**/exp OR **'shoulder prosthesis'**/exp OR **'conservative treatment'**/exp OR **'internal fixator'**/exp OR **'intramedullary nailing'**/exp OR **'plate fixation'**/exp OR **'bone screw'**/exp OR ((**conservative** OR **nonoperative** OR **'non operative'** OR **nonsurgical** OR **'non surgical'**) NEAR/3 (**management** OR **therapy** OR **treatment**)):ab,ti OR (**shoulder** NEAR/3 (**arthroplast*** OR **replacement*** OR **prosthes*** OR **endoprosthes***)):ab,ti OR ((**internal** OR **pin** OR **rod** OR **nail**) NEAR/3 (**fixation** OR **fixator***)):ab,ti OR ((**bone** OR **closed** OR **intramedullary** OR **medullary**) NEAR/3 (**nailing** OR **pinning** OR **plating**)):ab,ti OR (**plate*** NEAR/3 (**locking** OR **stabilization** OR **stabilization** OR **fixation** OR **ostheosynthesis** OR **philos** OR **lphp**)):ab,ti OR (**surgical** NEAR/3 (**management** OR **treatment** OR **therapy** OR **approach** OR **technique*** OR **procedure***)):ab,ti OR **surgery**:ab,ti	1,924,156
#1	**'proximal humerus fracture'**/exp OR (**'shoulder fracture'**/exp OR **'humerus fracture'**/exp AND (**proximal**:ab,ti OR **periprosth***:ab,ti OR **'peri implant*'**:ab,ti)) OR (**'periprosthetic fracture'**/exp AND (**humeral**:ab,ti OR **humerus**:ab,ti OR **shoulder***:ab,ti)) OR (((**proximal** OR **periprosth*** OR **'peri implant*'**) NEAR/6 **fracture***):ab,ti AND ((**humeral** OR **humerus** OR **shoulder**) NEAR/9 **fracture***):ab,ti)	3,215

Edit Email alert RSS feed

1,350 results for search #20 | Show all abstracts 1 — 25

Results View | Print | Export | Email | Order | Add to Clipboard

Select number of items Selected: 1350 (clear) Sort by: Relevance Publication Year Entry Date

1 Treatment of periprosthetic and peri-implant fractures: Modern plate osteosynthesis procedures

Raschke M.J., Stange R., Kösters C.
Chirurg 2012 83:8 (749-762)
MEDLINE Abstract Index Terms View Full Text

2 Internal fixation of proximal humerus fractures using the locking proximal humerus plate
Plecko M., Kraus A.
Operative Orthopadie und Traumatologie 2005 17:1 (25-50) Cited by: 103
Embase MEDLINE Abstract Index Terms View Full Text

3 Grammont reversed prosthesis for acute complex fracture of the proximal humerus in an elderly population with 5 to 12 years follow-up
Cazeneuve J.-F., Cristofari D.-J.
Revue de Chirurgie Orthopedique et Reparatrice de l'Appareil Moteur 2006 92:6 (543-548) Cited by: 69
Embase MEDLINE Abstract Index Terms View Full Text

4 Retrograde locked nailing of humeral shaft fractures: A prospective study of 58 cases
Apard T., Lahogue J.-F., Provė S., Hubert L., Talha A., Cronier P., Massin P.
Revue de Chirurgie Orthopedique et Reparatrice de l'Appareil Moteur 2006 92:1 (19-26) Cited by: 6
Embase MEDLINE Abstract Index Terms View Full Text

5 Medullary nailing in adult diaphyseal and proximal humeral fractures
Togninalli D., Remiger A.
Swiss Surgery 1998 4:4 (193-197) Cited by: 13
Embase MEDLINE Abstract Index Terms

6 Internal fixation of proximal humerus fracture by "palm tree" pinning
Le Bellec Y., Masmejean E., Cottias P., Alnot J.-Y., Huten D.
Revue de Chirurgie Orthopedique et Reparatrice de l'Appareil Moteur 2002 88:4 (342-348) Cited by: 17
Embase MEDLINE Abstract Index Terms

7 A new internal fixation device for proximal humeral fractures
Doursounian L., Grimberg J., Cazeau C., Touzard R.-C.
Revue de Chirurgie Orthopedique et Reparatrice de l'Appareil Moteur 1996 82:8 (743-752) Cited by: 22
Embase MEDLINE Abstract Index Terms

8 The interdisciplinary complications-conference - A simple method of quality management
Heim D., Stricker U., Negri M.
Swiss Surgery 2002 8:6 (243-249) Cited by: 1
Embase MEDLINE Abstract Index Terms View Full Text

9 Angle-stable intramedullary nailing of proximal humerus fractures with the PHN (Proximal Humeral Nail)
Blum J., Hansen M., Rommens P.M.
Operative Orthopadie und Traumatologie 2009 21:3 (296-311) Cited by: 17
Embase MEDLINE Abstract Index Terms View Full Text

10 Management of complications after angularly stable locking proximal humerus plate fixation
Voigt C., Woltmann A., Partenheimer A., Lill H.
Chirurg 2007 78:1 (40-46)
MEDLINE Abstract Index Terms View Full Text

11 Results after treatment of instable fractures of the proximal humerus using a fixed-angle plate
Hessler C., Schmucker U., Matthes G., Ekkernkamp A., Gütschow R., Eggers C.
Unfallchirurg 2006 109:10 (867-874) Cited by: 22
Embase MEDLINE Abstract Index Terms View Full Text

12 Shoulder arthroplasty for treatment of the sequelae of proximal humerus

fractures
Brunner U., Köhler S.
Orthopade 2007 36:11 (1037-1049) Cited by: 18
Embase MEDLINE Abstract Index Terms View Full Text

13 The cannulated blade plate 90° for displaced proximal humeral fractures in elderly patients
Fuchs M., Losch A., Stürmer K.M.
Zentralblatt fur Chirurgie 2003 128:1 (22-27) Cited by: 9
Embase MEDLINE Abstract Index Terms View Full Text

14 Results of shoulder arthroplasty for the treatment of late sequelae of humeral head fractures
Heitkemper S., Rickert M., Loew M.
Aktuelle Traumatologie 2004 34:3 (117-122) Cited by: 2
Embase Abstract Index Terms View Full Text

15 Primary endoprosthesis in comminuted fractures of the proximal - Humerus an alternative treatment for elderly patients?
Hoellen I.P., Bauer G., Holbein O.
Zentralblatt fur Chirurgie 1997 122:11 (994-1001) Cited by: 41
Embase MEDLINE Abstract Index Terms

16 Retrograde intramedullary wire-fixation of proximal humerus fractures in the elderly. Results of a prospectively documented study
Hoffmann R., Khodadadyan C., Raschke M., Melcher I., Maitino P.D., Haas N.P.
Zentralblatt fur Chirurgie 1998 123:11 (1232-1238) Cited by: 11
Embase MEDLINE Abstract Index Terms

17 Treatment of proximal humeral fractures with the PHILOS angular stable plate. Presentation of 225 cases of dislocated fractures
Kettler M., Biberthaler P., Braunstein V., Zeiler C., Kroetz M., Mutschler W.
Unfallchirurg 2006 109:12 (1032-1040) Cited by: 107
Embase MEDLINE Abstract Index Terms View Full Text

18 Osteoporosis is a risk factor for locking-plate implant failure of proximal humerus fractures
Haasters F., Kindsvater J., Siebenbürger G., Mutschler W., Ockert B.
Osteologie 2014 23:1 (16-21) Cited by: 1
Embase Abstract Index Terms

19 Humeral shaft fractures in the adult: Introduction
Lefèvre C.
Revue de Chirurgie Orthopedique et Reparatrice de l'Appareil Moteur 2004 90:5 SUPPL.(1S27-1S31) Cited by: 0
Embase Abstract Index Terms

20 Intramedullary fixation of humerus shaft fractures. An analysis of complications of 2 implants with special reference to outcome after management with the unreamed humerus interlocking nail
Vécsei N., Kolonja A., Mousavi M., Vécsei V.
Wiener klinische Wochenschrift 2001 113:15-16 (597-604) Cited by: 4
MEDLINE Abstract Index Terms

21 Reconstructive surgery following malunion of fractures of the proximal humerus in adults
Gerber C.
Der Orthopäde 1990 19:6 (316-323) Cited by: 13
MEDLINE Abstract Index Terms

22 Percutaneous osteosynthesis of humeral head fractures
Resch H., Hübner C., Aschauer E.
Operative Orthopadie und Traumatologie 2007 19:3 (276-293) Cited by: 6
Embase MEDLINE Abstract Index Terms View Full Text

23 Osteoporotic fractures of the proximal humerus
Lill H., Ellwein A., Katthagen C., Voigt C.
Chirurg 2012 83:10 (858-865)
MEDLINE Abstract Index Terms View Full Text

24 Minimally invasive internal fixation of proximal humeral fractures with a helix wire
Laminger K.A.
Operative Orthopadie und Traumatologie 2004 16:3 (253-272) Cited by: 0
Embase Abstract Index Terms View Full Text

25 Hemiarthroplasty for humeral head fractures
Wiedemann E., Brunner U., Hauptmann S., Mutschler W.
Operative Orthopadie und Traumatologie 2004 16:1 (1-27) Cited by: 4
Embase Abstract Index Terms View Full Text

Results View | Print | Export | Email | Order | Add to Clipboard

Records per page 25 Go to page: 1 of 54 Go

ELSEVIER

© 2016 RELX Intellectual Properties SA. All rights reserved.
Embase, RELX Group and the RE symbol are trade marks of RELX Intellectual Properties SA, used under license.

RELX Group™

Ovid®

Wolters Kluwer

Support & Training   Close

Database(s): **Ovid MEDLINE(R) In-Process & Other Non-Indexed Citations, Ovid MEDLINE(R) Daily and Ovid MEDLINE(R)** 1946 to Present

Search Strategy:

#	Searches	Results	Annotations
1	((exp Shoulder Fractures/ or exp Humeral Fractures/) and (proximal or periprosth* or peri-implant*).ab,ti.) or (Periprosthetic Fractures/ and (humeral or humerus or shoulder*).ab,ti.) or (((proximal or periprosth* or peri-implant*) adj6 fracture*) and ((humeral or humerus or shoulder) adj9 fracture*)).ab,ti.	2684	
2	exp Arthroplasty, Replacement/ or exp Joint Prosthesis/ or exp Fracture Fixation/ or exp internal fixators/ or Shoulder Joint/su or ((conservative or nonoperative or 'non operative' or nonsurgical or 'non surgical') adj3 (management or therapy or treatment)).ab,ti. or (shoulder adj3 (arthroplast* or replacement* or prosthes* or endoprosthes*)).ab,ti. or ((internal or pin or rod or nail) adj3 (fixation or fixator*)).ab,ti. or ((bone or closed or intramedullary or medullary) adj3 (nailing or pinning or plating)).ab,ti. or (plate* adj3 (locking or stabilization or stabilization or fixation or ostheosynthesis or philos or lphp)).ab,ti. or (surgical adj3 (management or treatment or therapy or approach or technique* or procedure*)).ab,ti. or surgery.ab,ti.	1230685	
3	1 and 2	2056	
4	Postoperative Complications/ or exp Arthroplasty/ae or Joint Prosthesis/ae or "Prostheses and Implants"/ae or exp Prosthesis Failure/ or complication*.ab,ti. or (adverse adj3 (effect* or event* or outcome or reaction*)).ab,ti. or (postoperative adj3 (complication* or pain or fail* or injur* or fractur*)).ab,ti. or ((persistent or movement or shoulder or syndrome) adj3 pain).ab,ti.	1230816	
5	Hematoma/ or exp Surgical Wound Infection/ or exp Bursitis/ or exp Shoulder Impingement Syndrome/ or Rotator Cuff/in or exp Femur Head Necrosis/ or exp Reoperation/ or Blood Loss, Surgical/ or exp Blood Transfusion/ or Pneumothorax/ or (infection or impingement or "frozen shoulder" or breakage or necrosis or osteonecrosis).ab,ti. or ((screw or wire) adj3 (perforation or cutout or cut-out or loosening or backing or penetration or perforation)).ab,ti. or ("rotator cuff" adj3 (lesion or injury or tendinopath* or disease or disorder* or symptom* or tear)).ab,ti. or ((implant or prosthes* or plate) adj5 (loosen* or tear* or wear or migration or subluxation or luxation or removal or displace* or fail* or dislocat* or instab* or malpositioning or loosening)).tw. or ((wound or implant or prosthetic) adj5 infect*).tw. or (septic adj5 (arthritis or prosthes*)).tw. or (osteolysis or impinge* or stiffness or hematoma* or haematoma* or thrombos* or impair* or palsy or palsies or disabilit* or mortality or death or dead or reoperation* or pneumothorax or refracture* or malunion or mal-union or nonunion or non-union or "fracture displacement" or "Scapular notching" or "Heterotopic ossification" or "Post-traumatic osteoarthritis" or "Posttraumatic osteoarthritis").ti,ab. or (blood adj3 (loss or transfusion)).ab,ti. or (fixation adj3 (problem* or loss)).ab,ti. or (tuberosity* adj3 (resorption or dislocation or migration or reduction or displacement)).ab,ti. or "glenoid erosion".ab,ti. or (gleno* adj3 degenerat*).ab,ti.	3026303	
6	4 or 5	3830423	
7	3 and 6	1410	
8	limit 7 to (english or french or german)	1302	
9	("11382420" or "19748802" or "19516088" or "18040648" or "21968245" or "24972435" or "21657970" or "23960366").ui.	8	
10	8 and 9	8	

1001. **Antegrade interlocking nailing of humeral shaft fractures.**

Petsatodes G; Karataglis D; Papadopoulos P; Christoforides J; Gigis J; Pournaras J.

Journal of Orthopaedic Science. 9(3):247-52, 2004.
[Journal Article]

UI: 15168178

Authors Full Name

Petsatodes, George; Karataglis, Dimitrios; Papadopoulos, Pericles; Christoforides, John; Gigis, John; Pournaras, John.

PubMed Advanced Search Builder

You Tube Tutorial

Use the builder below to create your search

Edit Clear

Builder

AND | All Fields | | Show index list

AND | All Fields | | Show index list

or Add to history

History

Download history Clear history

Search	Add to builder	Query	Items found	Time
#5	Add	Search **(((((((proximal[Title/Abstract] OR periprosth*[Title/Abstract] OR peri-implant*[Title/Abstract])) AND (humeral[Title/Abstract] OR humerus[Title/Abstract] OR shoulder[Title/Abstract])) AND fracture*[Title/Abstract])) AND (((inprocess[sb])) OR (publisher[sb] NOT pubstatusnihms NOT pubstatuspmcsd NOT pmcbook)))) AND ((conservative[Title/Abstract] OR nonoperative[Title/Abstract] OR "non operative"[Title/Abstract] OR nonsurgical[Title/Abstract] OR "non surgical" [Title/Abstract] OR arthroplast*[Title/Abstract] OR replacement*[Title/Abstract] OR prosthes*[Title/Abstract] OR endoprosthes*[Title/Abstract] OR pin[Title/Abstract] OR rod[Title/Abstract] OR nail[Title/Abstract] OR fixation[Title/Abstract] OR fixator*[Title/Abstract] OR nailing[Title/Abstract] OR pinning[Title/Abstract] OR plating[Title/Abstract] OR plate*[Title/Abstract] OR locking[Title/Abstract] OR stabilization[Title/Abstract] OR stabilization[Title/Abstract] OR fixation[Title/Abstract] OR ostheosynthesis[Title/Abstract] OR philos[Title/Abstract] OR lphp[Title/Abstract] OR surgical[Title/Abstract] OR surgery[Title/Abstract]))**	163	03:17:02
#4	Add	Search **(conservative[Title/Abstract] OR nonoperative[Title/Abstract] OR "non operative"[Title/Abstract] OR nonsurgical[Title/Abstract] OR "non surgical" [Title/Abstract] OR arthroplast*[Title/Abstract] OR replacement*[Title/Abstract] OR prosthes*[Title/Abstract] OR endoprosthes*[Title/Abstract] OR pin[Title/Abstract] OR rod[Title/Abstract] OR nail[Title/Abstract] OR fixation[Title/Abstract] OR fixator*[Title/Abstract] OR nailing[Title/Abstract] OR pinning[Title/Abstract] OR plating[Title/Abstract] OR plate*[Title/Abstract] OR locking[Title/Abstract] OR stabilization[Title/Abstract] OR stabilization[Title/Abstract] OR fixation[Title/Abstract] OR ostheosynthesis[Title/Abstract] OR philos[Title/Abstract] OR lphp[Title/Abstract] OR surgical[Title/Abstract] OR surgery[Title/Abstract])**	2295338	03:16:23
#3	Add	Search **(((((proximal[Title/Abstract] OR periprosth*[Title/Abstract] OR peri-implant*[Title/Abstract])) AND (humeral[Title/Abstract] OR humerus[Title/Abstract] OR shoulder[Title/Abstract])) AND fracture*[Title/Abstract])) AND (((inprocess[sb])) OR (publisher[sb] NOT pubstatusnihms NOT pubstatuspmcsd NOT pmcbook))**	201	03:12:00
#2	Add	Search **(((proximal[Title/Abstract] OR periprosth*[Title/Abstract] OR peri-implant*[Title/Abstract])) AND (humeral[Title/Abstract] OR humerus[Title/Abstract] OR shoulder[Title/Abstract])) AND fracture*[Title/Abstract]**	3040	03:11:51

#1	Add	Search **((inprocess[sb])) OR (publisher[sb] NOT pubstatusnihms NOT pubstatuspmcsd NOT pmcbook)** Sort by: **Relevance**	1088590	03:11:07

You are here: NCBI > Literature > PubMed

Write to the Help Desk

GETTING STARTED
NCBI Education
NCBI Help Manual
NCBI Handbook
Training & Tutorials
Submit Data

RESOURCES
Chemicals & Bioassays
Data & Software
DNA & RNA
Domains & Structures
Genes & Expression
Genetics & Medicine
Genomes & Maps
Homology
Literature
Proteins
Sequence Analysis
Taxonomy
Variation

POPULAR
PubMed
Bookshelf
PubMed Central
PubMed Health
BLAST
Nucleotide
Genome
SNP
Gene
Protein
PubChem

FEATURED
Genetic Testing Registry
PubMed Health
GenBank
Reference Sequences
Gene Expression Omnibus
Map Viewer
Human Genome
Mouse Genome
Influenza Virus
Primer-BLAST
Sequence Read Archive

NCBI INFORMATION
About NCBI
Research at NCBI
NCBI News
NCBI FTP Site
NCBI on Facebook
NCBI on Twitter
NCBI on YouTube

National Center for Biotechnology Information, U.S. National Library of Medicine
8600 Rockville Pike, Bethesda MD, 20894 USA
Policies and Guidelines | **Contact**

Scopus SciVal | Register Login Help

Brought to you by
UZH Hauptbibliothek / Zentralbibliothek Zürich

Search Alerts Lists My Scopus

Register now for the free Scopus webinar on July 21st: Learn about what types of APIs Scopus offers and how they can benefit you

Document search | Author search | Affiliation search | Advanced search Browse Sources Compare journals

Search tips Field codes

TITLE-ABS-KEY (complication* OR (adverse W/3 (effect* OR event* OR outcome OR reaction*)) OR (postoperative W/3 (complication* or pain or fail* or injur* or fractur*)) OR ((persistent OR movement OR shoulder OR syndrome) W/3 pain) OR (infection OR impingement OR "frozen shoulder" OR breakage OR necrosis) OR ((screw OR wire) W/3 (perforation OR cutout OR cut-out OR loosening OR backing)) OR ("rotator cuff" W/3 (lesion OR injury OR tendinopath* OR disease OR disorder* OR symptom* OR tear)) OR ((implant or prosthes* OR plate) W/5 (loosen* or tear* or wear or migration or subluxation or luxation or removal or displace* or fail* or dislocat* or instab* OR malpositioning OR loosening)) OR ((wound or implant or prosthetic) W/5 infect*) OR (septic W/5 (arthritis or prosthes*)) OR (osteolysis or impinge* or stiffness or hematoma* or haematoma* or thrombos* or impair* or palsy or palsies OR disabilit* or mortality OR death OR dead OR reoperation* OR pneumothorax OR refracture* OR malunion OR mal-union OR nonunion OR non-union OR "fracture displacement" OR "Scapular notching" OR "Heterotopic ossification" OR "Post-traumatic osteoarthritis" OR "Posttraumatic osteoarthritis") OR (blood W/3 (loss or transfusion)) OR (fixation W/3 (problem* OR loss)) OR (tuberosity* W/3 (resorption OR dislocation OR migration OR reduction OR displacement)) OR "glenoid erosion" OR (gleno* W/3 degenerat*))

Outline query | Clear form | Add Author name / Affiliation

As you type Scopus offers code suggestions. Double click or press "enter" to add to advanced search.

AND
OR
AND NOT
PRE/
W/
ABS
AF-ID
AFFIL
AFFILCITY
AFFILCOUNTRY
AFFILORG
ALL
ARTNUM
AU-ID

Advanced search examples:
ALL("**heart attack**") AND AUTHOR-NAME(**smith**)
TITLE-ABS-KEY(***somatic complaint wom?n**) AND PUBYEAR AFT **1993**
SRCTITLE(***field ornith***) AND VOLUME(**75**) AND ISSUE(**1**) AND PAGES(**53-66**)

Search history

6 (TITLE-ABS-KEY (((**proximal** OR **periprosth*** OR **peri-implant***) W/6 **fracture***) AND ((**humeral** OR **humerus** OR **shoulder**) W/9 **fracture***))) AND (TITLE-ABS-KEY (((**conservative** OR **nonoperative** OR **"non operative"** OR **nonsurgical** OR **"non surgical"**) W/3 (**management** OR **therapy** OR **treatment**)) OR (**shoulder** W/3 (**arthroplast*** OR **replacement*** OR **prosthes*** OR **endoprosthes***)) OR ((**internal** OR **pin** OR **rod** OR **nail**) W/3 (**fixation** OR **fixator***)) OR ((**bone** OR **closed** OR **intramedullary** OR **medullary**) W/3 (**nailing** OR **pinning** OR **plating**)) OR (**plate*** W/3 (**locking** OR **stabilization** OR **stabilization** OR **fixation** OR **ostheosynthesis** OR **philos** OR **lphp**)) OR (**surgical** W/3 (**management** OR **treatment** OR **therapy** OR **approach** OR **technique*** OR **procedure***)) OR **surgery**)) AND (TITLE-ABS-KEY (**complication*** OR (**adverse** W/3 (**effect*** OR **event*** OR **outcome** OR **reaction***)) OR (**postoperative** W/3 (**complication*** OR **pain** OR **fail*** OR **injur*** OR **fractur***)) OR ((**persistent** OR **movement** OR **shoulder** OR **syndrome**) W/3 **pain**) OR (**infection** OR **impingement** OR **"frozen shoulder"** OR **breakage** OR **necrosis**) OR ((**screw** OR **wire**) W/3 (**perforation** OR **cutout** OR **cut-out** OR **loosening** OR **backing**)) OR (**"rotator cuff"** W/3 (**lesion** OR **injury** OR **tendinopath*** OR **disease** OR **disorder*** OR **symptom*** OR **tear**)) OR ((**implant** OR **prosthes*** OR **plate**) W/5 (**loosen*** OR **tear*** OR **wear** OR **migration** OR **subluxation** OR **luxation** OR **removal** OR **displace*** OR **fail*** OR **dislocat*** OR **instab*** OR **malpositioning** OR **loosening**)) OR ((**wound** OR **implant** OR **prosthetic**) W/5 **infect***) OR (**septic** W/5 (**arthritis** OR **prosthes***)) OR (**osteolysis** OR **impinge*** OR **stiffness** OR **hematoma*** OR **haematoma*** OR **thrombos*** OR **impair*** OR **palsy** OR **palsies** OR **disabilit*** OR **mortality** OR **death** OR **dead** OR **reoperation*** OR **pneumothorax** OR **refracture*** OR **malunion** OR **mal-union** OR **nonunion** OR **non-union** OR **"fracture displacement"** OR **"Scapular notching"** OR **"Heterotopic ossification"** OR **"Post-traumatic osteoarthritis"** OR **"Posttraumatic osteoarthritis"**) OR (**blood** W/3 (**loss** OR **transfusion**)) OR (**fixation** W/3 (**problem*** OR **loss**)) OR (**tuberosity*** W/3 (**resorption** OR **dislocation** OR **migration** OR **reduction** OR **displacement**)) OR **"glenoid erosion"** OR (**gleno*** W/3 **degenerat***))) AND (LIMIT-TO (LANGUAGE , **"English"**) OR LIMIT-TO (LANGUAGE , **"German"**) OR LIMIT-TO (LANGUAGE , **"French"**)) — 1,576 document results

5 TITLE-ABS-KEY (((**proximal** OR **periprosth*** OR **peri-implant***) W/6 **fracture***) AND ((**humeral** OR **humerus** OR **shoulder**) W/9 **fracture***))) AND (TITLE-ABS-KEY (((**conservative** OR **nonoperative** OR **"non operative"** OR **nonsurgical** OR **"non surgical"**) W/3 (**management** OR **therapy** OR **treatment**)) OR (**shoulder** W/3 (**arthroplast*** OR **replacement*** OR **prosthes*** OR **endoprosthes***)) OR ((**internal** OR **pin** OR **rod** OR **nail**) W/3 (**fixation** OR **fixator***)) OR ((**bone** OR **closed** OR **intramedullary** OR **medullary**) W/3 (**nailing** OR **pinning** OR **plating**)) OR (**plate*** W/3 (**locking** OR **stabilization** OR **stabilization** OR **fixation** OR **ostheosynthesis** OR **philos** OR **lphp**)) OR (**surgical** W/3 (**management** OR **treatment** OR **therapy** OR **approach** OR **technique*** OR **procedure***)) OR **surgery**)) AND (TITLE-ABS-KEY (**complication*** OR (**adverse** W/3 (**effect*** OR **event*** OR **outcome** OR **reaction***)) OR (**postoperative** W/3 (**complication*** OR **pain** OR **fail*** OR **injur*** OR **fractur***)) OR ((**persistent** OR **movement** OR **shoulder** OR **syndrome**) W/3 **pain**) OR (**infection** OR **impingement** OR **"frozen shoulder"** OR **breakage** OR **necrosis**) OR ((**screw** OR **wire**) W/3 (**perforation** OR **cutout** OR **cut-out** OR **loosening** OR **backing**)) OR (**"rotator cuff"** W/3 (**lesion** OR **injury** OR **tendinopath*** OR **disease** OR **disorder*** OR **symptom*** OR **tear**)) OR ((**implant** OR **prosthes*** OR **plate**) W/5 (**loosen*** OR **tear*** OR **wear** OR **migration** OR **subluxation** OR **luxation** OR **removal** OR **displace*** OR **fail*** OR **dislocat*** OR **instab*** OR **malpositioning** OR **loosening**)) OR ((**wound** OR **implant** OR **prosthetic**) W/5 **infect***) OR (**septic** W/5 (**arthritis** OR **prosthes***)) OR (**osteolysis** OR **impinge*** OR **stiffness** OR **hematoma*** OR **haematoma*** OR **thrombos*** OR **impair*** OR **palsy** OR **palsies** OR **disabilit*** OR **mortality** OR **death** OR **dead** OR **reoperation*** OR **pneumothorax** OR **refracture*** OR **malunion** OR **mal-union** OR **nonunion** OR **non-union** OR **"fracture displacement"** OR **"Scapular notching"** OR **"Heterotopic ossification"** OR **"Post-traumatic osteoarthritis"** OR **"Posttraumatic osteoarthritis"**) OR (**blood** W/3 (**loss** OR **transfusion**)) OR (**fixation** W/3 (**problem*** OR **loss**)) OR (**tuberosity*** W/3 (**resorption** OR **dislocation** OR **migration** OR **reduction** OR **displacement**)) OR **"glenoid erosion"** OR (**gleno*** W/3 **degenerat***))) — 1,730 document results

4 TITLE-ABS-KEY (**complication*** OR (**adverse** W/3 (**effect*** OR **event*** OR **outcome** OR **reaction***)) OR (**postoperative** W/3 (**complication*** OR **pain** OR **fail*** OR **injur*** OR **fractur***)) OR ((**persistent** OR **movement** OR **shoulder** OR **syndrome**) W/3 **pain**) OR (**infection** OR **impingement** OR **"frozen shoulder"** OR **breakage** OR **necrosis**) OR ((**screw** OR **wire**) W/3 (**perforation** OR **cutout** OR **cut-out** OR **loosening** OR **backing**)) OR (**"rotator cuff"** W/3 (**lesion** OR **injury** OR **tendinopath*** OR **disease** OR **disorder*** OR **symptom*** OR **tear**)) OR ((**implant** OR **prosthes*** OR **plate**) W/5 (**loosen*** OR **tear*** OR **wear** OR **migration** OR **subluxation** OR **luxation** OR **removal** OR **displace*** OR **fail*** OR **dislocat*** OR **instab*** OR **malpositioning** OR **loosening**)) OR ((**wound** OR **implant** OR **prosthetic**) W/5 **infect***) OR (**septic** W/5 (**arthritis** OR **prosthes***)) OR (**osteolysis** OR **impinge*** OR **stiffness** OR **hematoma*** OR **haematoma*** OR **thrombos*** OR **impair*** OR **palsy** OR **palsies** OR **disabilit*** OR **mortality** OR **death** OR **dead** OR **reoperation*** OR **pneumothorax** OR **refracture*** OR **malunion** OR **mal-union** OR **nonunion** OR **non-union** OR **"fracture displacement"** OR **"Scapular notching"** OR **"Heterotopic ossification"** OR **"Post-traumatic osteoarthritis"** OR **"Posttraumatic osteoarthritis"**) OR (**blood** W/3 (**loss** OR **transfusion**)) OR (**fixation** W/3 (**problem*** OR **loss**)) OR (**tuberosity*** W/3 (**resorption** OR **dislocation** OR **migration** OR **reduction** OR **displacement**)) OR **"glenoid erosion"** OR (**gleno*** W/3 **degenerat***)) — 7,100,315 document results

3 TITLE-ABS-KEY (((**conservative** OR **nonoperative** OR **"non operative"** OR **nonsurgical** OR **"non surgical"**) W/3 (**management** OR **therapy** OR **treatment**)) OR (**shoulder** W/3 (**arthroplast*** OR **replacement*** OR **prosthes*** OR **endoprosthes***)) OR ((**internal** OR **pin** OR **rod** OR **nail**) W/3 (**fixation** OR **fixator***)) OR ((**bone** OR **closed** OR **intramedullary** OR **medullary**) W/3 (**nailing** OR **pinning** OR **plating**)) OR (**plate*** W/3 (**locking** OR **stabilization** OR **stabilization** OR **fixation** OR **ostheosynthesis** OR **philos** OR **lphp**)) OR (**surgical** W/3 (**management** OR **treatment** OR **therapy** OR **approach** OR **technique*** OR **procedure***)) OR **surgery**) — 2,219,224 document results

1 TITLE-ABS-KEY (((**proximal** OR **periprosth*** OR **peri-implant***) W/6 **fracture***) AND ((**humeral** OR **humerus** OR **shoulder**) W/9 **fracture***)) — 3,075 document results

Top of page

Copyright © 2016 Elsevier B.V. All rights reserved. Scopus® is a registered trademark of Elsevier B.V.
Cookies are set by this site. To decline them or learn more, visit our Cookies page

RELX Group

Wiley Online Library

Cochrane Library
Trusted evidence.
Informed decisions.
Better health.

Logged In: **Martina Gosteli**
My Profile Institutional Login Log Out

Search | Search Manager | Medical Terms (MeSH) | Browse

To search an exact word(s) use quotation marks, e.g. "hospital" finds hospital; hospital (no quotation marks) finds hospital and hospitals; pay finds paid, pays, paying, payed)

Add to top

View fewer lines

#1	((proximal or periprosth* or peri-implant*) near/6 fracture*) and ((humeral or humerus or shoulder) near/9 fracture*):ti,ab,kw (Word variations have been searched)	0	139
#2	((conservative or nonoperative or 'non operative' or nonsurgical or 'non surgical') near/3 (management or therapy or treatment)) or (shoulder near/3 (arthroplast* or replacement* or prosthes* or endoprosthes*)) or ((internal or pin or rod or nail) near/3 (fixation or fixator*)) or ((bone or closed or intramedullary or medullary) near/3 (nailing or pinning or plating)) or (plate* near/3 (locking or stabilization or stabilization or fixation or ostheosynthesis or philos or lphp)) or (surgical near/3 (management or treatment or therapy or approach or technique* or procedure*)) or surgery:ti,ab,kw (Word variations have been searched)	1	105181
#3	complication* or (adverse near/3 (effect* or event* or outcome or reaction*)) or (postoperative near/3 (complication* or pain or fail* or injur* or fractur*)) or ((persistent or movement or shoulder or syndrome) near/3 pain) or (infection or impingement or "frozen shoulder" or breakage or necrosis) or ((screw or wire) near/3 (perforation or cutout or cut-out or loosening or backing)) or ("rotator cuff" near/3 (lesion or injury or tendinopath* or disease or disorder* or symptom* or tear)) or ((implant or prosthes* or plate) near/5 (loosen* or tear* or wear or migration or subluxation or luxation or removal or displace* or fail* or dislocat* or instab* or malpositioning or loosening)) or ((wound or implant or prosthetic) near/5 infect*) or (septic near/5 (arthritis or prosthes*)) or (osteolysis or impinge* or stiffness or hematoma* or haematoma* or thrombos* or impair* or palsy or palsies or disabilit* or mortality or death or dead or reoperation* or pneumothorax or refracture* or malunion or mal-union or nonunion or non-union or "fracture displacement" or "Scapular notching" or "Heterotopic ossification" or "Post-traumatic osteoarthritis" or "Posttraumatic osteoarthritis") or (blood near/3 (loss or transfusion)) or (fixation near/3 (problem* or loss)) or (tuberosity* near/3 (resorption or dislocation or migration or reduction or displacement)) or "glenoid erosion" or (gleno* near/3 degenerat*):ti,ab,kw (Word variations have been searched)	2	304854
#4		3	75
#5		4	N/A

Clear Strategy Search Help

Highlight orphan lines

Save strategy

Strategy Name

Comments

Save Strategy

Strategy Library (124)

All Results (75)

- Cochrane Reviews (1)
  - All
  - Review
  - Protocol
- Other Reviews (2)
- Trials (71)
- Methods Studies (0)
- Technology Assessments (0)
- Economic Evaluations (1)
- Cochrane Groups (0)

- All
- Current Issue

Me Methodology
Dx Diagnostic
Ov Overview

Cochrane Database of Systematic Reviews : Issue 7 of 12, July 2016

Issue ***updated daily*** throughout month

There is **1** result from **9474** records for your search on **#4 - #1 and #2 and #3** in **Cochrane Reviews** in the strategy currently being edited

Sort by Relevance: high to low

Select all | Export all Export selected

FREE Interventions for treating **proximal humeral fractures** in adults
Helen HG Handoll and Stig Brorson
Online Publication Date: November 2015

Ns Cc Review

Export selected

Pg Prognosis

Qu Qualitative

Cc Conclusions changed

Ns New search

Mc Major change

Up Update

Wd Withdrawn

Cm Comment

Wiley Online Library

Publications Browse By Subject Resources

About us Help Contact Us Agents Advertisers Media Privacy Cookies Terms & Conditions Site Map

Copyright © 1999–2016 John Wiley & Sons, Inc. All Rights Reserved.

About Wiley Wiley.com Wiley Job Network WILEY

Search Results for *Proximal Humerus Fractures – update 2017/07/07*
Search protocols:

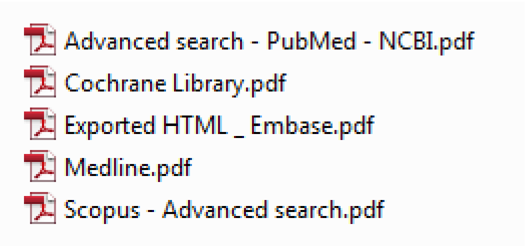

	References	References after Deduplication
**EMBASE**	198	133
**Medline**	92	87
**PubMed**	186	106
**Scopus**	149	35
**Cochrane**	29*	17
**Pool**	654	378

Reference files: Proximal_humerus_fractures_update.enlx
Cochrane: neue Treffer durch Deduplizierung mit ursprünglicher Cochrane-Suche, (siehe Screenshots Seite 2)

before deduplication:

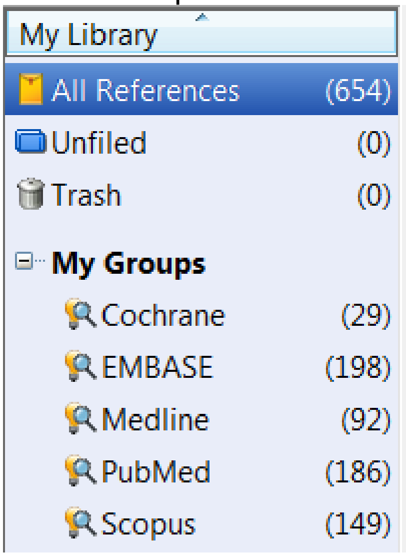

after:

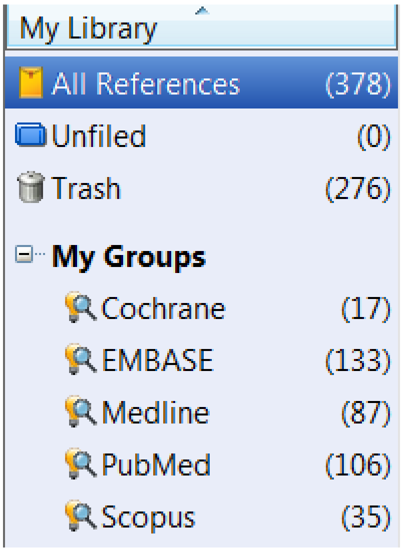

Cochrane
before:

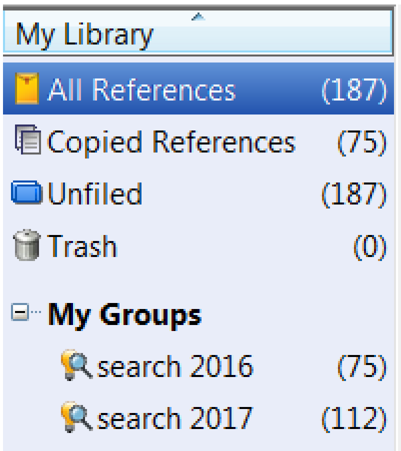

after

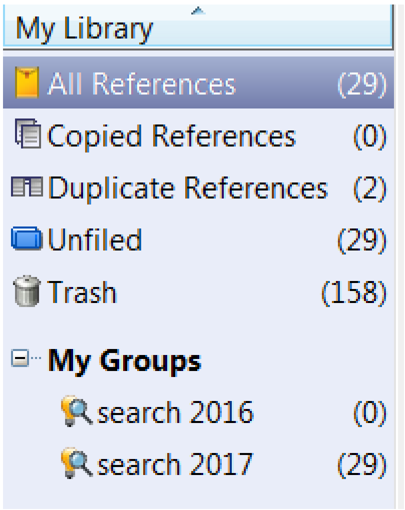

Embase®

Embase Session Results (7 Jul 2017)

No.	Query	Results
#9	#3 AND #6 NOT [conference abstract]/lim AND ([english]/lim OR [french]/lim OR [german]/lim) AND [18-7-2016]/sd NOT [7-7-2017]/sd	198
#8	#3 AND #6 NOT [conference abstract]/lim AND ([english]/lim OR [french]/lim OR [german]/lim)	1540
#7	#3 AND #6	1806
#6	#4 OR #5	5481468
#5	'hematoma'/exp OR 'infection rate'/exp OR 'frozen shoulder'/exp OR 'shoulder impingement syndrome'/exp OR 'rotator cuff injury'/exp OR 'neurological complication'/exp OR 'femur head necrosis'/exp OR 'screw loosening'/exp OR 'reoperation'/exp OR 'operative blood loss'/exp OR 'postoperative hemorrhage'/exp OR 'blood transfusion'/exp OR 'pneumothorax'/exp OR infection:ab,ti OR impingement:ab,ti OR 'frozen shoulder':ab,ti OR breakage:ab,ti OR necrosis:ab,ti OR ((screw OR wire) NEAR/3 (perforation OR cutout OR 'cut out' OR loosening OR backing)):ab,ti OR ('rotator cuff' NEAR/3 (lesion OR injury OR tendinopath* OR disease OR disorder* OR symptom* OR tear)):ab,ti OR ((implant OR prosthes* OR plate) NEAR/5 (loosen* OR tear* OR wear OR migration OR subluxation OR luxation OR removal OR displace* OR fail* OR dislocat* OR instab* OR malpositioning OR loosening)):ab,ti OR ((wound OR implant OR prosthetic) NEAR/5 infect*):ab,ti OR (septic NEAR/5 (arthritis OR prosthes*)):ab,ti OR osteolysis:ab,ti OR impinge*:ab,ti OR stiffness:ab,ti OR hematoma*:ab,ti OR haematoma*:ab,ti OR thrombos*:ab,ti OR impair*:ab,ti OR palsy:ab,ti OR palsies:ab,ti OR disabilit*:ab,ti OR mortality:ab,ti OR death:ab,ti OR dead:ab,ti OR reoperation*:ab,ti OR pneumothorax:ab,ti OR refracture*:ab,ti OR malunion:ab,ti OR 'mal union':ab,ti OR nonunion:ab,ti OR 'non union':ab,ti OR 'fracture displacement':ab,ti OR 'scapular notching':ab,ti OR 'heterotopic ossification':ab,ti OR 'post-traumatic osteoarthritis':ab,ti OR 'posttraumatic osteoarthritis':ab,ti OR (blood NEAR/3 (loss OR transfusion)):ab,ti OR (fixation NEAR/3 (problem* OR loss)):ab,ti OR (tuberosity* NEAR/3 (resorption OR dislocation OR migration OR reduction OR displacement)):ab,ti OR 'glenoid erosion':ab,ti OR (gleno* NEAR/3 degenerat*):ab,ti	4214539
#4	'adverse outcome'/exp OR 'treatment failure'/exp OR 'postoperative complication'/exp OR complication*:ab,ti OR (adverse NEAR/3 (effect* OR event* OR outcome OR reaction*)):ab,ti OR (postoperative NEAR/3 (complication* OR pain OR fail* OR injur* OR fractur*)):ab,ti OR ((persistent OR movement OR shoulder OR syndrome) NEAR/3 pain):ab,ti	1990060
#3	#1 AND #2	2517
#2	'shoulder arthroplasty'/exp OR 'shoulder prosthesis'/exp OR 'conservative treatment'/exp OR 'internal fixator'/exp OR 'intramedullary nailing'/exp OR 'plate fixation'/exp OR 'bone screw'/exp OR ((conservative OR nonoperative OR 'non operative' OR nonsurgical OR 'non surgical') NEAR/3 (management OR therapy OR treatment)):ab,ti OR (shoulder NEAR/3 (arthroplast* OR replacement* OR prosthes* OR endoprosthes*)):ab,ti OR ((internal OR pin OR rod OR nail) NEAR/3 (fixation OR fixator*)):ab,ti OR ((bone OR closed OR intramedullary OR medullary) NEAR/3 (nailing OR pinning OR plating)):ab,ti OR (plate* NEAR/3 (locking OR stabilization OR stabilization OR fixation OR ostheosynthesis OR philos OR lphp)):ab,ti OR (surgical NEAR/3 (management OR treatment OR therapy OR approach OR technique* OR procedure*)):ab,ti OR surgery:ab,ti	2066166
#1	'proximal humerus fracture'/exp OR ('shoulder fracture'/exp OR 'humerus fracture'/exp AND (proximal:ab,ti OR periprosth*:ab,ti OR 'peri implant*':ab,ti)) OR ('periprosthetic fracture'/exp AND (humeral:ab,ti OR humerus:ab,ti OR shoulder*:ab,ti)) OR (((proximal OR periprosth* OR 'peri implant*') NEAR/6 fracture*):ab,ti AND ((humeral OR humerus OR shoulder) NEAR/9 fracture*):ab,ti)	3658

ELSEVIER

© 2017 RELX Intellectual Properties SA. All rights reserved.
Embase, RELX Group and the RE symbol are trade marks of RELX Intellectual Properties SA, used under license.

Database(s): **Ovid MEDLINE(R) In-Process & Other Non-Indexed Citations, Ovid MEDLINE(R) Daily and Ovid MEDLINE(R)** 1946 to Present

Search Strategy:

#	Searches	Results
1	((exp Shoulder Fractures/ or exp Humeral Fractures/) and (proximal or periprosth* or peri-implant*).ab,ti.) or (Periprosthetic Fractures/ and (humeral or humerus or shoulder*).ab,ti.) or (((proximal or periprosth* or periimplant*) adj6 fracture*) and ((humeral or humerus or shoulder) adj9 fracture*)).ab,ti.	2892
2	exp Arthroplasty, Replacement/ or exp Joint Prosthesis/ or exp Fracture Fixation/ or exp internal fixators/ or Shoulder Joint/su or ((conservative or nonoperative or 'non operative' or nonsurgical or 'non surgical') adj3 (management or therapy or treatment)).ab,ti. or (shoulder adj3 (arthroplast* or replacement* or prosthes* or endoprosthes*)).ab,ti. or ((internal or pin or rod or nail) adj3 (fixation or fixator*)).ab,ti. or ((bone or closed or intramedullary or medullary) adj3 (nailing or pinning or plating)).ab,ti. or (plate* adj3 (locking or stabilization or stabilization or fixation or ostheosynthesis or philos or lphp)).ab,ti. or (surgical adj3 (management or treatment or therapy or approach or technique* or procedure*)).ab,ti. or surgery.ab,ti.	1319214
3	1 and 2	2220
4	Postoperative Complications/ or exp Arthroplasty/ae or Joint Prosthesis/ae or "Prostheses and Implants"/ae or exp Prosthesis Failure/ or complication*.ab,ti. or (adverse adj3 (effect* or event* or outcome or reaction*)).ab,ti. or (postoperative adj3 (complication* or pain or fail* or injur* or fractur*)).ab,ti. or ((persistent or movement or shoulder or syndrome) adj3 pain).ab,ti.	1332967
5	Hematoma/ or exp Surgical Wound Infection/ or exp Bursitis/ or exp Shoulder Impingement Syndrome/ or Rotator Cuff/in or exp Femur Head Necrosis/ or exp Reoperation/ or Blood Loss, Surgical/ or exp Blood Transfusion/ or Pneumothorax/ or (infection or impingement or "frozen shoulder" or breakage or necrosis or osteonecrosis).ab,ti. or ((screw or wire) adj3 (perforation or cutout or cut-out or loosening or backing or penetration or perforation)).ab,ti. or ("rotator cuff" adj3 (lesion or injury or tendinopath* or disease or disorder* or symptom* or tear)).ab,ti. or ((implant or prosthes* or plate) adj5 (loosen* or tear* or wear or migration or subluxation or luxation or removal or displace* or fail* or dislocat* or instab* or malpositioning or loosening)).tw. or ((wound or implant or prosthetic) adj5 infect*).tw. or (septic adj5 (arthritis or prosthes*)).tw. or (osteolysis or impinge* or stiffness or hematoma* or haematoma* or thrombos* or impair* or palsy or palsies or disabilit* or mortality or death or dead or reoperation* or pneumothorax or refracture* or malunion or mal-union or nonunion or non-union or "fracture displacement" or "Scapular notching" or "Heterotopic ossification" or "Post-traumatic osteoarthritis" or "Posttraumatic osteoarthritis").ti,ab. or (blood adj3 (loss or transfusion)).ab,ti. or (fixation adj3 (problem* or loss)).ab,ti. or (tuberosity* adj3 (resorption or dislocation or migration or reduction or displacement)).ab,ti. or "glenoid erosion".ab,ti. or (gleno* adj3 degenerat*).ab,ti.	3259680
6	4 or 5	4129312
7	3 and 6	1517
8	limit 7 to (english or french or german)	1400
9	(2016 07* or 2016 08* or 2016 09* or 2016 10* or 2016 11* or 2016 12* or 2017*).dt.	985507
10	8 and 9	92

1. Transhumeral Anterior Radial Nerve Transposition to Simplify Anticipated Future **Humeral** Reconstruction. [Review]

   Muzykewicz DA; Abrams RA.

   Journal of Hand Surgery - American Volume. 42(7):578.e1-578.e5, 2017 Jul.

   [Journal Article. Review]

   UI: 28526166

   Authors Full Name
   Muzykewicz, David A; Abrams, Reid A.

chur_test My NCBI Sign Out

NCBI Resources How To

PubMed Home | More Resources | Help

PubMed Advanced Search Builder

YouTube Tutorial

Use the builder below to create your search

Edit Clear

Builder

AND All Fields Show index list

AND All Fields Show index list

or Add to history

History

Download history Clear history

Search	Add to builder	Query	Items found	Time
#2	Add	Search **((((((proximal[Title/Abstract] OR periprosth*[Title/Abstract] OR periimplant*[Title/Abstract])) AND (humeral[Title/Abstract] OR humerus[Title/Abstract] OR shoulder[Title/Abstract])) AND fracture*[Title/Abstract])) AND (((inprocess[sb])) OR (publisher[sb] NOT pubstatusnihms NOT pubstatuspmcsd NOT pmcbook)))) AND ((conservative[Title/Abstract] OR nonoperative[Title/Abstract] OR "non operative"[Title/Abstract] OR nonsurgical[Title/Abstract] OR "non surgical" [Title/Abstract] OR arthroplast*[Title/Abstract] OR replacement*[Title/Abstract] OR prosthes*[Title/Abstract] OR endoprosthes*[Title/Abstract] OR pin[Title/Abstract] OR rod[Title/Abstract] OR nail[Title/Abstract] OR fixation[Title/Abstract] OR fixator*[Title/Abstract] OR nailing[Title/Abstract] OR pinning[Title/Abstract] OR plating[Title/Abstract] OR plate*[Title/Abstract] OR locking[Title/Abstract] OR stabilization[Title/Abstract] OR stabilization[Title/Abstract] OR fixation[Title/Abstract] OR ostheosynthesis[Title/Abstract] OR philos[Title/Abstract] OR lphp[Title/Abstract] OR surgical[Title/Abstract] OR surgery[Title/Abstract]))**	186	03:55:03

You are here: NCBI > Literature > PubMed Support Center

GETTING STARTED
NCBI Education
NCBI Help Manual
NCBI Handbook
Training & Tutorials
Submit Data

RESOURCES
Chemicals & Bioassays
Data & Software
DNA & RNA
Domains & Structures
Genes & Expression
Genetics & Medicine
Genomes & Maps
Homology
Literature
Proteins

POPULAR
PubMed
Bookshelf
PubMed Central
PubMed Health
BLAST
Nucleotide
Genome
SNP
Gene
Protein

FEATURED
Genetic Testing Registry
PubMed Health
GenBank
Reference Sequences
Gene Expression Omnibus
Map Viewer
Human Genome
Mouse Genome
Influenza Virus
Primer-BLAST

NCBI INFORMATION
About NCBI
Research at NCBI
NCBI News & Blog
NCBI FTP Site
NCBI on Facebook
NCBI on Twitter
NCBI on YouTube

Scopus

Search Sources Alerts Lists SciVal Register Login

Advanced search

Compare sources

Documents Authors Affiliations Advanced

Search tips

Enter query string

(TITLE-ABS-KEY(((proximal OR periprosth*) W/6 fracture*) AND ((humeral OR humerus OR shoulder) W/9 fracture*)) OR TITLE-ABS-KEY(((peri-implant*) W/6 fracture*) AND ((humeral OR humerus OR shoulder) W/9 fracture*))) AND (TITLE-ABS-KEY (((conservative OR nonoperative OR "non operative" OR nonsurgical OR "non surgical") W/3 (management OR therapy OR treatment)) OR (shoulder W/3 (arthroplast* OR replacement* OR prosthes* OR endoprosthes*)) OR ((internal OR pin OR rod OR nail) W/3 (fixation OR fixator*)) OR ((bone OR closed OR intramedullary OR medullary) W/3 (nailing OR pinning OR plating)) OR (plate* W/3 (locking OR stabilization OR stabilization OR fixation OR ostheosynthesis OR philos OR lphp)) OR (surgical W/3 (management OR treatment OR therapy OR approach OR technique* OR procedure*)) OR surgery)) AND (TITLE-ABS-KEY (complication* OR (adverse W/3 (effect* OR event* OR outcome OR reaction*)) OR (postoperative W/3 (complication* or pain or fail* or injur* or fractur*)) OR ((persistent OR movement OR shoulder OR syndrome) W/3 pain) OR (infection OR impingement OR "frozen shoulder" OR breakage OR necrosis) OR ((screw OR wire) W/3 (perforation OR cutout OR cut-out OR loosening OR backing)) OR ("rotator cuff" W/3 (lesion OR injury OR tendinopath* OR disease OR disorder* OR symptom* OR tear)) OR ((implant or prosthes* OR plate) W/5 (loosen* or tear* or wear or migration or subluxation or luxation or removal or displace* or fail* or dislocat* or instab* OR malpositioning OR loosening)) OR ((wound or

Operators

AND

OR

AND NOT

PRE/

W/

Field codes

ABS

AF-ID

AFFIL

implant or prosthetic) W/5 infect*) OR (septic W/5 (arthritis or prosthes*)) OR (osteolysis or impinge* or stiffness or hematoma* or haematoma* or thrombos* or impair* or palsy or palsies OR disabilit* or mortality OR death OR dead OR reoperation* OR pneumothorax OR refracture* OR malunion OR mal-union OR nonunion OR non-union OR "fracture displacement" OR "Scapular notching" OR "Heterotopic ossification" OR "Post-traumatic osteoarthritis" OR "Posttraumatic osteoarthritis") OR (blood W/3 (loss or transfusion)) OR (fixation W/3 (problem* OR loss)) OR (tuberosity* W/3 (resorption OR dislocation OR migration OR reduction OR displacement)) OR "glenoid erosion" OR (gleno* W/3 degenerat*))) AND (LIMIT-TO (LANGUAGE,"English") OR LIMIT-TO (LANGUAGE,"German") OR LIMIT-TO (LANGUAGE,"French")) AND ORIG-LOAD-DATE > 20160718

AFFILCITY

AFFILCOUNTRY

AFFILORG

ALL

ARTNUM

AU-ID

AUTH

ALL("heart attack") AND AUTHOR-NAME(smith)

*TITLE-ABS-KEY(*somatic complaint wom?n) AND PUBYEAR AFT 1993*

*SRCTITLE(*field ornith*) AND VOLUME(75) AND ISSUE(1) AND PAGES(53-66)*

Operators and field codes can be added by typing it in the query field, clicking on the "+" icon or by clicking on the "add" button in the example pop out.

Outline query Add Author name / Affiliation Clear form

Search history

6 (TITLE-ABS-KEY (((proximal OR periprosth*) W/6 fracture*) AND ((humeral OR humerus OR shoulder) W/9 fracture*)) OR TITLE-ABS-KEY (((peri-implant*) W/6 fracture*) AND ((humeral OR humerus OR shoulder) W/9 fracture*))) AND (TITLE-ABS-KEY (((conservative OR nonoperative OR "non operative" OR nonsurgical OR "non surgical") W/3 (management OR therapy C... 149 document results

5 (TITLE-ABS-KEY (((proximal OR periprosth*) W/6 fracture*) AND ((humeral OR humerus OR shoulder) W/9 fracture*)) OR TITLE-ABS-KEY (((peri-implant*) W/6 fracture*) AND ((humeral OR humerus OR shoulder) W/9 fracture*))) AND (TITLE-ABS-KEY (((conservative OR nonoperative OR "non operative" OR nonsurgical OR "non surgical") W/3 (management OR therapy C... 1,752 document results

4 (TITLE-ABS-KEY (((proximal OR periprosth*) W/6 fracture*) AND ((humeral OR humerus OR shoulder) W/9 fracture*)) OR TITLE-ABS-KEY (((peri-implant*) W/6 fracture*) AND ((humeral OR humerus OR shoulder) W/9 fracture*))) AND 1,915 document results

(TITLE-ABS-KEY (((conservative OR nonoperative OR "non operative" OR nonsurgical OR "non surgical") W/3 (management OR therapy C···

3 TITLE-ABS-KEY (complication* OR (adverse W/3 (effect* OR event* OR outcome OR reaction*)) OR (postoperative W/3 (complication* OR pain OR fail* OR injur* OR fractur*)) OR ((persistent OR movement OR shoulder OR syndrome) W/3 pain) OR (infection OR impingement OR "frozen shoulder" OR breakage OR necrosis) OR ((screw OR wire) W/3 (perforation OR cutou··· — 7,574,872 document results

2 TITLE-ABS-KEY (((conservative OR nonoperative OR "non operative" OR nonsurgical OR "non surgical") W/3 (management OR therapy OR treatment)) OR (shoulder W/3 (arthroplast* OR replacement* OR prosthes* OR endoprosthes*)) OR ((internal OR pin OR rod OR nail) W/3 (fixation OR fixator*)) OR ((bone OR closed OR intramedullary OR medullar··· — 2,345,621 document results

Showing 5 most recent searches | View all 6

Top of page

Brought to you by
UZH Hauptbibliothek / Zentralbibliothek Zürich

Wiley Online Library

Cochrane Library

Trusted evidence.
Informed decisions.
Better health.

Logged In: **Martina Gosteli**
My Profile Institutional Login Log Out

Search | Search Manager | Medical Terms (MeSH) | Browse

To search an exact word(s) use quotation marks, e.g. "hospital" finds hospital; hospital (no quotation marks) finds hospital and hospitals; pay finds paid, pays, paying, payed)

Add to top

View fewer lines

#1	((proximal or periprosth* or peri-implant*) near/6 fracture*) and ((humeral or humerus or shoulder) near/9 fracture*):ti,ab,kw	185
#2	((conservative or nonoperative or 'non operative' or nonsurgical or 'non surgical') near/3 (management or therapy or treatment)) or (shoulder near/3 (arthroplast* or replacement* or prosthes* or endoprosthes*)) or ((internal or pin or rod or nail) near/3 (fixation or fixator*)) or ((bone or closed or intramedullary or medullary) near/3 (nailing or pinning or plating)) or (plate* near/3 (locking or stabilization or stabilization or fixation or ostheosynthesis or philos or lphp)) or (surgical near/3 (management or treatment or therapy or approach or technique* or procedure*)) or surgery:ti,ab,kw	126732
#3	complication* or (adverse near/3 (effect* or event* or outcome or reaction*)) or (postoperative near/3 (complication* or pain or fail* or injur* or fractur*)) or ((persistent or movement or shoulder or syndrome) near/3 pain) or (infection or impingement or "frozen shoulder" or breakage or necrosis) or ((screw or wire) near/3 (perforation or cutout or cut-out or loosening or backing)) or ("rotator cuff" near/3 (lesion or injury or tendinopath* or disease or disorder* or symptom* or tear)) or ((implant or prosthes* or plate) near/5 (loosen* or tear* or wear or migration or subluxation or luxation or removal or displace* or fail* or dislocat* or instab* or malpositioning or loosening)) or ((wound or implant or prosthetic) near/5 infect*) or (septic near/5 (arthritis or prosthes*)) or (osteolysis or impinge* or stiffness or hematoma* or haematoma* or thrombos* or impair* or palsy or palsies or disabilit* or mortality or death or dead or reoperation* or pneumothorax or refracture* or malunion or mal-union or nonunion or non-union or "fracture displacement" or "Scapular notching" or "Heterotopic ossification" or "Post-traumatic osteoarthritis" or "Posttraumatic osteoarthritis") or (blood near/3 (loss or transfusion)) or (fixation near/3 (problem* or loss)) or (tuberosity* near/3 (resorption or dislocation or migration or reduction or displacement)) or "glenoid erosion" or (gleno* near/3 degenerat*):ti,ab,kw	450446
#4	#1 and #2 and #3	112
#5	#1 and #2 and #3<br>Publication Year from 2016 to 2017	31
#6		N/A

Clear Strategy Search Help

Highlight orphan lines

Save strategy

Strategy Name Save Strategy

Comments

▶ Strategy Library (131)

All Results (112)

- Cochrane Reviews (5)
  - All
  - Review
  - Protocol
- Other Reviews (4)
- Trials (102)
- Methods Studies (0)
- Technology Assessments (0)
- Economic Evaluations (1)
- Cochrane Groups (0)

NHS Economic Evaluation Database : Issue 2 of 4, April 2015

There is **1** result from **17433** records for your search on **#4** - **#1 and #2 and #3** in **Economic Evaluations** in the strategy currently being edited

Sort by Relevance: high to low

Select all | Export all | Export selected

Functional results of angular-stable **plate fixation** in **displaced proximal humeral fractures** (Provisional abstract)
Centre for Reviews and Dissemination
Original Author(s): Handschin A E , Cardell M , Contaldo C , Trentz O and Wanner G A
Injury, 2008, 39, 306-313

Export selected

Me	Methodology
Dx	Diagnostic
Ov	Overview
Pg	Prognosis
Qu	Qualitative
Cc	Conclusions changed
Ns	New search
Mc	Major change
Up	Update
Wd	Withdrawn
Cm	Comment

Copyright © 1999–2017 John Wiley & Sons, Inc. All Rights Reserved.
