# Supplementary material for: Complications after surgical management of proximal humeral fractures: a systematic review of event terms and definitions
Source: BMC Musculoskelet Disord. 2020 May 26;21:327. doi: 10.1186/s12891-020-03353-8 (PMC7251821; doi:10.1186/s12891-020-03353-8)
Supplement: Supplementary file 2 — Additional file 2. List of reviewed scientific articles and book chapters. [file 12891_2020_3353_MOESM2_ESM.pdf]

Supplementary file 2

**Article title**	Complications after surgical management of proximal humeral fractures: a systematic review of event terms and definitions
**Journal name**	BMC Musculoskeletal Disorders
**Author names**	Alispahic N, Brorson S, Bahrs C, Joeris A, Steinitz A, Audigé L
**Corresponding author**	Laurent Audigé, Schulthess Klinik, CH-8008 Zurich, Switzerland
**e-mail address**	laurent.audige@kws.ch

List of reviewed scientific articles and book chapters

Scientific articles

1. Agarwal S, Rana A, Sharma RK. Functional outcome after primary hemiarthroplasty in three or four part proximal humerus fracture: A short term followup. Indian J Orthop 2016;50:590-594. doi: 10.4103/0019-5413.193481
2. Aguado HJ, Mingo J, Torres M, Alvarez-Ramos A, Martin-Ferrero MA. Minimally invasive polyaxial locking plate osteosynthesis for 3-4 part proximal humeral fractures: our institutional experience. Injury 2016;47 Suppl 3:S22-S28. doi: https://dx.doi.org/10.1016/S0020-1383(16)30602-7
3. Altintas B, Biber R, Bail HJ. Is it safe to assist proximal humeral nailing to residents? An analysis of 1134 cases. Injury 2016;47 Suppl 7:S7-S9. doi: https://dx.doi.org/10.1016/S0020-1383(16)30846-4
4. Ayoub MA, Gad H, El-Tantawy A, Atef A, Seleem OA. Geriatric complex proximal humeral fracture: Intraoperative locking plate problems and proposed solutions. Current Orthopaedic Practice 2017;28:70-78. doi: 10.1097/BCO.0000000000000456
5. Bahrs C, Kuhle L, Blumenstock G, Stockle U, Rolauffs B, Freude T. Which parameters affect medium- to long-term results after angular stable plate fixation for proximal humeral fractures? Journal of Shoulder & Elbow Surgery. 2015;24(5):727-732. PMID: 25441560
6. Beirer M, Crönlein M, Venjakob AJ, et al. Additional calcar support using a blade device reduces secondary varus displacement following reconstruction of the proximal humerus: A prospective study. European Journal of Medical Research. 2015;20(1). PMID:
7. Biazzo A, Cardile C, Brunelli L, Ragni P, Clementi D. Early results for treatment of two- and three-part fractures of the proximal humerus using Contours PHP (proximal humeral plate). Acta Biomed Ateneo Parmense 2017;88:65-73. doi: https://dx.doi.org/10.23750/abm.v88i1.5193
8. Bockmann B, Buecking B, Franz D, Zettl R, Ruchholtz S, Mohr J. Mid-term results of a less-invasive locking plate fixation method for proximal humeral fractures: a prospective observational study. BMC Musculoskeletal Disorders. 2015;16:160. PMID: 26141352
9. Bogdan Y, Gausden EB, Zbeda R, Helfet DL, Lorich DG, Wellman DS. An alternative technique for greater tuberosity fractures: use of the mesh plate. Arch Orthop Trauma Surg 2017. doi: 10.1007/s00402-017-2715-x
10. Bonnevialle N, Tournier C, Clavert P, Ohl X, Sirveaux F, Saragaglia D et al. Hemiarthroplasty versus reverse shoulder arthroplasty in 4-part displaced fractures of the proximal humerus: Multicenter retrospective study. Orthopaedics and Traumatology: Surgery and Research 2016;102:569-573. doi: 10.1016/j.otsr.2016.02.014

11. Boyer E, Menu G, Loisel F, Saadnia R, Uhring J, Adam A et al. Cementless and locked prosthesis for the treatment of 3-part and 4-part proximal humerus fractures: prospective clinical evaluation of hemi- and reverse arthroplasty. European Journal of Orthopaedic Surgery and Traumatology 2017;27:301-308. doi: 10.1007/s00590-017-1926-8
12. Caceres-Sanchez L, Mesa-Mateo A, Barrionuevo-Sanchez FJ, Garcia-Benitez B, Exposito-Triano S. [Total reverse shoulder replacement. Evaluation of the clinical results and complications in a series of 52 cases]. Rev Esp Cir Ortop Traumatol. 2015;59(6):439-446. PMID: 26004517
13. Chandrappa MH, Hajibandeh S, Hajibandeh S. Postoperative outcomes of initial varus versus initial valgus proximal humerus fracture: A systematic review and meta-analysis. Journal of Clinical Orthopaedics & Trauma 2017;8:14-20. doi: https://dx.doi.org/10.1016/j.jcot.2016.09.011
14. Chen H, Hu X, Tang H, Yang G, Xiang M. Minimal Invasive Percutaneous Osteosynthesis for Elderly Valgus Impacted Proximal Humeral Fractures with the PHILOS. BioMed Research International. 2015;2015:971216. PMID: 26693491
15. Cho CH, Kim DH, Kim BS. Radiographic and clinical results of tension suture fixation using two washers with PHILOS plate for proximal humeral fractures. Injury 2017;48:464-468. doi: https://dx.doi.org/10.1016/j.injury.2016.11.030
16. Christiano A, Pean C, Konda S, Egol K. Functional outcome after proximal humerus fracture fixation : Understanding the risk factors. Acta Orthop Belg 2017;83:1-7. doi:
17. Chun YM, Kim DS, Lee DH, Shin SJ. Reverse shoulder arthroplasty for four-part proximal humerus fracture in elderly patients: can a healed tuberosity improve the functional outcomes? J Shoulder Elbow Surg 2017;26:1216-1221. doi: https://dx.doi.org/10.1016/j.jse.2016.11.034
18. Cicak N, Klobucar H, Medancic N. Reverse shoulder arthroplasty in acute fractures provides better results than in revision procedures for fracture sequelae.[Erratum appears in Int Orthop. 2015 Feb;39(2):377 Note: Nikola, Cicak [corrected to Cicak, Nikola]; Hrvoje, Klobucar [corrected to Klobucar, Hrvoje]; Nenad, Medancic [corrected to Medancic, Nenad]; PMID: 25576249]. International Orthopaedics. 2015;39(2):343-348. PMID: 25548128
19. Cuny C, Goetzmann T, Dedome D, et al. Antegrade nailing evolution for proximal humeral fractures, the Telegraph IV(): a study of 67 patients. European journal of orthopaedic surgery & traumatologie. 2015;25(2):287-295. PMID: 24947347
20. Cuny C. Proximal humerus fractures: editorial. Eur 2017;27:279-284. doi: https://dx.doi.org/10.1007/s00590-017-1928-6
21. D'Ambrosi R, Palumbo F, Barbato A, Facchini RM. A prospective study for the treatment of proximal humeral fractures with the Galaxy Fixation System. Musculoskelet Surg 2017;101:11-17. doi: 10.1007/s12306-016-0434-z
22. Fang C, Kwek EBK. Self-reducing proximal humerus fractures. J 2017;25:2309499017717180. doi: https://dx.doi.org/10.1177/2309499017717180
23. Fattoretto D, Borgo A, Iacobellis C. The treatment of complex proximal humeral fractures: analysis of the results of 55 cases treated with PHILOS plate. Musculoskelet Surg 2016;100:109-114. doi: 10.1007/s12306-015-0395-7
24. Gadea F, Favard L, Boileau P, Cuny C, d'Ollone T, Saragaglia D et al. Fixation of 4-part fractures of the proximal humerus: Can we identify radiological criteria that support locking plates or IM nailing? Comparative, retrospective study of 107 cases. Orthop Traumatol Surg Res 2016;102:963-970. doi: https://dx.doi.org/10.1016/j.otsr.2016.09.015
25. Garofalo R, Flanagin B, Castagna A, Lo EY, Krishnan SG. Long stem reverse shoulder arthroplasty and cerclage for treatment of complex long segment proximal humeral fractures with diaphyseal extension in patients more than 65 years old. Injury. 2015;46(12):2379-2383. PMID: 26521994
26. Garret J, Houdre H, Cievet-Bonfils M, Godeneche A, Duparc F, Roussignol X. Surgical treatment of complex proximal humeral fractures with a technique of nail and osteosuture: 'NOS'. Eur 2017;27:391-397. doi: https://dx.doi.org/10.1007/s00590-017-1939-3

27. Gavaskar AS, Karthik BB, Tummala NC, Srinivasan P, Gopalan H. Second generation locked plating for complex proximal humerus fractures in very elderly patients. Injury 2016;47:2534-2538. doi: https://dx.doi.org/10.1016/j.injury.2016.08.010

28. Gigis I, Nenopoulos A, Giannekas D, Heikenfeld R, Beslikas T, Hatzokos I. Reverse Shoulder Arthroplasty for the Treatment of 3 and 4- Part Fractures of the Humeral Head in the Elderly. Open Orthop J 2017;11:108-118. doi: https://dx.doi.org/10.2174/1874325001711010108

29. Goch AM, Christiano A, Konda SR, Leucht P, Egol KA. Operative repair of proximal humerus fractures in septuagenarians and octogenarians: Does chronologic age matter? Journal of Clinical Orthopaedics & Trauma 2017;8:50-53. doi: https://dx.doi.org/10.1016/j.jcot.2017.01.006

30. Gonc U, Atabek M, Teker K, Tanriover A. Minimally invasive plate osteosynthesis with PHILOS plate for proximal humerus fractures. Acta Orthop Traumatol Turc 2017;51:17-22. doi: https://dx.doi.org/10.1016/j.aott.2016.10.003

31. Gracitelli M, Malavolta E, Assuncao J, Kojima K, Dos RP, Silva J et al. Locking intramedullary nails compared with locking plates for two- and threepart proximal humeral surgical neck fractures: a randomized controlled trial. In. Journal of shoulder and elbow surgery Conference: ASES 2016 annual meeting United states; 2017, p. e149.

32. Greiwe RM. Proximal humerus fractures: Percutaneous fixation, proximal humeral nailing, and open reduction and internal fixation. In. Shoulder and Elbow Trauma and its Complications: Elsevier Inc.; 2015. p. 83-112. (ISBN No. 9781782424727 (ISBN); 9781782424499 (ISBN))

33. Grubhofer F, Wieser K, Meyer DC, Catanzaro S, Beeler S, Riede U et al. Reverse total shoulder arthroplasty for acute head-splitting, 3- and 4-part fractures of the proximal humerus in the elderly. Journal of Shoulder and Elbow Surgery 2016;25:1690-1698. doi: 10.1016/j.jse.2016.02.024

34. Gumina S, Baudi P, Candela V, Campochiaro G. Hertel 7 fracture of the humeral head. Can two different fixation systems (Diphos/PHP) lead to different outcomes? A retrospective study. Injury 2016;47 Suppl 4:S59-S63. doi: https://dx.doi.org/10.1016/j.injury.2016.07.051

35. Haasters F, Siebenbürger G, Helfen T, Daferner M, Böcker W, Ockert B. Complications of locked plating for proximal humeral fractures-are we getting any better? Journal of Shoulder and Elbow Surgery 2016;25:e295-e303. doi: 10.1016/j.jse.2016.02.015

36. Hageman MGJS, Meijer D, Stufkens SA, Ring D, Doornberg JN, Steller EP. Proximal humeral fractures: Nonoperative versus operative treatment. Archives of Trauma Research 2017;6. doi: 10.5812/atr.37423

37. Handoll Helen HG, Brorson S. Interventions for treating proximal humeral fractures in adults. Cochrane Database of Systematic Reviews: John Wiley & Sons, Ltd; 2015.

38. Handoll HH, Keding A, Corbacho B, Brealey SD, Hewitt C, Rangan A. Five-year follow-up results of the PROFHER trial comparing operative and non-operative treatment of adults with a displaced fracture of the proximal humerus. Bone Joint J 2017;99-B:383-392. doi: https://dx.doi.org/10.1302/0301-620X.99B3.BJJ-2016-1028

39. Hao TD, Huat AWT. Surgical technique and early outcomes of intramedullary nailing of displaced proximal humeral fractures in an Asian population using a contemporary straight nail design. J 2017;25:2309499017713934. doi: https://dx.doi.org/10.1177/2309499017713934

40. Hashmi FR, Mayr E. A new nail with a locking blade for complex proximal humeral fractures. European Journal of Orthopaedic Surgery and Traumatology 2016;26:831-836. doi: 10.1007/s00590-016-1817-4

41. Hernandez-Elena J, de la Red-Gallego MA, Garces-Zarzalejo C, et al. [Treatment of proximal humeral fractures by reverse shoulder arthroplasty: mid-term evaluation of functional results and Notching]. Rev Esp Cir Ortop Traumatol. 2015;59(6):413-420. PMID: 26165592

42. Imrecke J, Katthagen JC, Lill H. Fracture endoprostheses of the shoulder: When and how? Trauma und Berufskrankheit. 2015;17(3):160-165. PMID:

43. Inui H, Nobuhara K. Assessment of the restriction of arm elevation after intramedullary fixation for proximal humeral fractures. Shoulder & elbow 2017;9:100-104. doi: 10.1177/1758573216687265
44. Kanchanatawan W, Suppauksorn S, Chobpenthai T, Densiri-aksorn W, Pongpinyopap W, Dorjiee G. Surgical Technique for Open Reduction-Internal Fixation of an Unstable Displaced 3-Part Proximal Humeral Fracture Using a Proximal Humeral Locking Plate. Arthroscopy Techniques 2017. doi: 10.1016/j.eats.2017.02.008
45. Kancherla VK, Singh A, Anakwenze OA. Management of Acute Proximal Humeral Fractures. J Am Acad Orthop Surg 2017;25:42-52. doi: 10.5435/jaaos-d-15-00240
46. Katthagen JC, Ellwein A, Lutz O, Voigt C, Lill H. Outcomes of proximal humeral fracture fixation with locked CFR-PEEK plating. Eur 2017;27:351-358. doi: https://dx.doi.org/10.1007/s00590-016-1891-7
47. Koljonen PA, Fang C, Lau TW, Leung F, Cheung NW. Minimally invasive plate osteosynthesis for proximal humeral fractures. Journal of Orthopaedic Surgery. 2015;23(2):160-163. PMID: 26321541
48. Konigshausen M, Thierbach A, Kubler L, et al. [Surgical treatment of 3- and 4-part fractures of the humeral head using a polyaxial-locking plate: results and patient satisfaction]. Zeitschrift fur Orthopadie & Unfallchirurgie. 2015;153(1):51-58. PMID: 25723581
49. Kovalak E, Atay T, Baykal YB, Başal Ö. Surgical management of 3 and 4-part proximal humerus fractures with locking plates in elderly. Journal of Clinical and Analytical Medicine 2017;8:243-247. doi: 10.4328/JCAM.4834
50. Lange M, Brandt D, Mittlmeier T, Gradl G. Proximal humeral fractures: non-operative treatment versus intramedullary nailing in 2-, 3- and 4-part fractures. Injury 2016;47 Suppl 7:S14-S19. doi: https://dx.doi.org/10.1016/S0020-1383(16)30848-8
51. Liu YW, Wei XE, Kuang Y, Zheng YX, Gu XF, Zhan HS et al. Open vs. closed reduction combined with minimally invasive plate osteosynthesis in humeral fractures. Minim Invasive Ther Allied Technol 2016;25:215-221. doi: 10.3109/13645706.2016.1151891
52. Longo UG, Petrillo S, Berton A, Denaro V. Reverse total shoulder arthroplasty for the management of fractures of the proximal humerus: a systematic review. Musculoskelet Surg 2016;100:83-91. doi: 10.1007/s12306-016-0409-0
53. Lowry V, Bureau NJ, Desmeules F, Roy JS, Rouleau DM. Acute proximal humeral fractures in adults. J Hand Ther 2017;30:158-166. doi: 10.1016/j.jht.2017.05.005
54. Merschin D, Stangl R. Proximal humeral fractures in the elderly: Quality of life, clinical results and institutionalization following primary reverse fracture arthroplasty. Unfallchirurg. 2015. PMID:
55. Miyazaki AN, Santos PD, Sella GD, Duarte DC, Giunta GD, Checchia SL. Outcomes of non-arthroplasty surgical treatment of proximal humeral head fractures. Rev 2016;51:527-534. doi: https://dx.doi.org/10.1016/j.rboe.2016.08.011
56. Muhm M, Bott J, Lahr C, Winkler H, Ruffing T. Outcome after operative treatment of proximal humeral fractures in elderly patients. Zeitschrift fur Gerontologie und Geriatrie. 2015. PMID:
57. Neviaser RJ. Proximal humeral locking plates for displaced fractures of the proximal humeral humerus. Proximal Humerus Fractures: Evaluation and Management: Springer International Publishing; 2015:99-105.
58. Obert L, Saadnia R, Tournier C, Bonnevialle N, Saragaglia D, Sirveaux F. Four-part fractures treated with a reversed total shoulder prosthesis: Prospective and retrospective multicenter study. Results and complications. Orthop Traumatol Surg Res 2016;102:279-285. doi: 10.1016/j.otsr.2016.01.019

59. Okike K, Lee OC, Makanji H, Morgan JH, Harris MB, Vrahas MS. Comparison of locked plate fixation and nonoperative management for displaced proximal humerus fractures in elderly patients. American Journal of Orthopedics (Chatham, Nj). 2015;44(4):E106-112. PMID: 25844592

60. Ortmaier R, Mattiassich G, Pumberger M, et al. Comparison between reverse shoulder arthroplasty and Humerusblock in three- and four-part proximal humerus fractures in elderly patients. International Orthopaedics. 2015;39(2):335-342. PMID: 25038969

61. Papakonstantinou MK, Hart MJ, Farrugia R, Gosling C, Kamali Moaveni A, van Bavel D et al. Prevalence of non-union and delayed union in proximal humeral fractures. ANZ J Surg 2017;87:55-59. doi: https://dx.doi.org/10.1111/ans.13756

62. Parada SA, Makani A, Stadecker MJ, Warner JJ. Technique of Open Reduction and Internal Fixation of Comminuted Proximal Humerus Fractures With Allograft Femoral Head Metaphyseal Reconstruction. American Journal of Orthopedics (Chatham, Nj). 2015;44(10):471-475. PMID: 26447409

63. Park YK, Kim SH, Oh JH. Intermediate-term outcome of hemiarthroplasty for comminuted proximal humerus fractures. J Shoulder Elbow Surg 2017;26:85-91. doi: https://dx.doi.org/10.1016/j.jse.2016.05.008

64. Patel S, Colaco HB, Elvey ME, Lee MH. Post-traumatic osteonecrosis of the proximal humerus. Injury. 2015;46(10):1878-1884. PMID:

65. Repetto I, Alessio-Mazzola M, Cerruti P, Sanguineti F, Formica M, Felli L. Surgical management of complex proximal humeral fractures: pinning, locked plate and arthroplasty : Clinical results and functional outcome on retrospective series of patients. Musculoskelet Surg 2017. doi: 10.1007/s12306-017-0451-6

66. Roberson TA, Granade CM, Hunt Q, Griscom JT, Adams KJ, Momaya AM et al. Nonoperative management versus reverse shoulder arthroplasty for treatment of 3- and 4-part proximal humeral fractures in older adults. Journal of Shoulder and Elbow Surgery 2017;26:1017-1022. doi: 10.1016/j.jse.2016.10.013

67. Rotini R, Cavaciocchi M, Fabbri D, et al. Proximal humeral fracture fixation: multicenter study with carbon fiber peek plate. Musculoskeletal Surgery. 2015;99 Suppl 1:S1-8. PMID: 25962808

68. Sabharwal S, Patel NK, Griffiths D, Athanasiou T, Gupte CM, Reilly P. Trials based on specific fracture configuration and surgical procedures likely to be more relevant for decision making in the management of fractures of the proximal humerus: Findings of a meta-analysis. Bone Joint Res 2016;5:470-480. doi: https://dx.doi.org/10.1302/2046-3758.510.2000638

69. Schairer WW, Nwachukwu BU, Lyman S, Craig EV, Gulotta LV. Reverse shoulder arthroplasty versus hemiarthroplasty for treatment of proximal humerus fractures. Journal of Shoulder & Elbow Surgery. 2015;24(10):1560-1566. PMID: 25958208

70. Schnetzke M, Bockmeyer J, Porschke F, Studier-Fischer S, Grutzner PA, Guehring T. Quality of Reduction Influences Outcome After Locked-Plate Fixation of Proximal Humeral Type-C Fractures. J Bone Joint Surg Am 2016;98:1777-1785. doi: https://dx.doi.org/10.2106/JBJS.16.00112

71. Sethi PM, MacKen CJ. Management of Greater Tuberosity Fractures. Techniques in Shoulder and Elbow Surgery 2016;17:102-109. doi: 10.1097/BTE.0000000000000089

72. Sharaby MMF. Results of biological restoration of varus impacted proximal humeral fracture and stabilization with locked plate and calcar screws. Current Orthopaedic Practice 2016;27:524-529. doi: 10.1097/BCO.0000000000000416

73. Shukla DR, McAnany S, Pean C, Overley S, Lovy A, Parsons BO. The results of tension band rotator cuff suture fixation of locked plating of displaced proximal humerus fractures. Injury 2017;48:474-480. doi: https://dx.doi.org/10.1016/j.injury.2016.12.022

74. Sinha N, Rao BS, Trivedy PD, Rao AS. Letter to the Editor: Minimally invasive plate osteosynthesis for proximal humeral fractures. J Orthop Surg (Hong Kong) 2016;24:139. doi: 10.1177/230949901602400140

75. Sohn HS, Jeon YS, Lee J, Shin SJ. Clinical comparison between open plating and minimally invasive plate osteosynthesis for displaced proximal humeral fractures: A prospective randomized controlled trial. Injury 2017;48:1175-1182. doi: 10.1016/j.injury.2017.03.027

76. Solomon JA, Joseph SM, Shishani Y, Victoroff BN, Wilber JH, Gobezie R et al. Cost analysis of hemiarthroplasty versus reverse shoulder arthroplasty for fractures. Orthopedics 2016;39:230-234. doi: 10.3928/01477447-20160610-03

77. Trikha V, Singh V, Choudhury B, Das S. Retrospective analysis of proximal humeral fracture-dislocations managed with locked plates. Journal of Shoulder and Elbow Surgery 2017. doi: 10.1016/j.jse.2017.03.035

78. Ueyama H, Yano K, Kanemura M, Gotani H, Ito S, Sakanaka H. Clinical outcomes and prognostic factors depending on implant design in the treatment of proximal humeral fractures: A retrospective study. Journal of Orthopaedics 2016;13:369-375. doi: 10.1016/j.jor.2016.06.025

79. Uzer G, Yildiz F, Batar S, Binlaksar R, Elmadag M, Kus G et al. Does grafting of the tuberosities improve the functional outcomes of proximal humeral fractures treated with reverse shoulder arthroplasty? J Shoulder Elbow Surg 2017;26:36-41. doi: https://dx.doi.org/10.1016/j.jse.2016.05.005

80. Valenti P, Aliani D, Maroun C, Werthel JD, Elkolti K. Shoulder hemiarthroplasty for proximal humeral fractures: analysis of clinical and radiographic outcomes at midterm follow-up: a series of 51 patients. Eur J Orthop Surg Traumatol 2017;27:309-315. doi: 10.1007/s00590-017-1927-7

81. van der Merwe M, Boyle MJ, Frampton CMA, Ball CM. Reverse shoulder arthroplasty compared with hemiarthroplasty in the treatment of acute proximal humeral fractures. J Shoulder Elbow Surg 2017. doi: 10.1016/j.jse.2017.02.005

82. Vijayvargiya M, Pathak A, Gaur S. Outcome Analysis of Locking Plate Fixation in Proximal Humerus Fracture. J Clin Diagn Res 2016;10:RC01-05. doi: https://dx.doi.org/10.7860/JCDR/2016/18122.8281

83. Villodre-Jimenez J, Estrems-Diaz V, Diranzo-Garcia J, Bru-Pomer A. Reverse shoulder arthroplasty in 3 and 4 part proximal humeral fractures in patients aged more than 65 years: Results and complications. Revista espanola de cirugia ortopedica y traumatologia 2017;61:43-50. doi: https://dx.doi.org/10.1016/j.recot.2016.09.005

84. Westphal T, Woischnik S, Adolf D, Feistner H, Piatek S. Axillary nerve lesions after open reduction and internal fixation of proximal humeral fractures through an extended lateral deltoid-split approach: electrophysiological findings. J Shoulder Elbow Surg 2017;26:464-471. doi: https://dx.doi.org/10.1016/j.jse.2016.07.027

85. White JJE, Soothill JR, Morgan M, Clark DI, Espag MP, Tambe AA. Outcomes for a large metaphyseal volume hemiarthroplasty in complex fractures of the proximal humerus. Journal of Shoulder and Elbow Surgery 2017;26:478-483. doi: 10.1016/j.jse.2016.08.004

86. Willert T, Schmidt T, Gehring J, Weber J, Westphal T. Reversed total shoulder arthroplasty for proximal humerus fractures : Health-related quality of life. Obere Extremitat 2017:1-6. doi: 10.1007/s11678-017-0410-8

87. Wolfensperger F, Grüninger P, Dietrich M, Völlink M, Benninger E, Schläppi M et al. Reverse shoulder arthroplasty for complex fractures of the proximal humerus in elderly patients: Impact on the level of independency, early function, and pain medication. Journal of Shoulder and Elbow Surgery 2017. doi: 10.1016/j.jse.2017.01.021

88. Xia S, Zhang Y, Wang X, et al. Computerized Virtual Surgery Planning for ORIF of Proximal Humeral Fractures. Orthopedics. 2015;38(5):e428-433. PMID: 25970372

89. Zacharia B, Puthezhath K, Varghees I. Kirschner wire migration from subcapital humeral fracture site, causing hydropneumothorax. Chin J Traumatol 2016;19:305-308. doi:

90. Zhao L, Yang P, Zhu L, Chen AM. Minimal invasive percutaneous plate osteosynthesis (MIPPO) through deltoid-pectoralis approach for the treatment of elderly proximal humeral fractures. BMC Musculoskelet Disord 2017;18:187. doi: https://dx.doi.org/10.1186/s12891-017-1538-9
91. Zirngibl B, Biber R, Bail HJ. Humeral head necrosis after proximal humeral nailing: what are the reasons for bad outcomes? Injury 2016;47:S10-S13. doi: 10.1016/S0020-1383(16)30847-6

Book chapters

92. Baker M, Choi K, Ghasemi SR, McFarland EG. Reverse Shoulder Arthroplasty. In: Huri G, Paschos N, editors. The Shoulder. Cham: Springer; 2017. p. 293-349.
93. Bohsali, K., A. Bois, and M. Wirth, Fractures of the Proximal Humerus, in Rockwood and Matsen's the shoulder, C.A. Rockwood, et al., Editors. 2017, Elsevier, Inc. p. 183-242.
94. Brorson, S. (2017). Proximal humeral fractures. in: Musculoskeletal trauma in the elderly. M. McQueen, A. Charles Court-Brown, D. Duckworth et al. Boca Raton, CRC Press.
95. Brunner U. (2017) Kopferhaltende Therapie der proximalen Humerusfraktur, in: Schulterchirurgie, Habermeyer P., Lichtenberg S., Loew M., Magosch P., Martetschläger F., Tauber M. (eds), 5th Edition, Elsevier GmbH, München, p484-534
96. Chunyan Jiang (2017) Humerus, proximal, in: AO Principles of Fracture Management, Third Edition, edited by Buckley R., Moran C., Apivatthakakul T, 587-606
97. Ellwein, A., et al. (2017). Plattenosteosynthese bei proximaler Humerusfraktur. in: Schulter. L.-J. Lehmann and M. Loew, Springer-Verlag GmbH: 179-187.
98. Esenyel CZ. Proximal Humerus Fractures. In: Huri G, Paschos NK, editors. The shoulder; 2017. (ISBN No. ISBN 978-3-319-51979-1)
99. Lehmann, L.-J. and D. Rzepka (2017). Anatomische Hemiendoprothetik und inverse Totalendoprothetik bei proximaler Humerusfraktur. in: Schulter. L.-J. Lehmann and M. Loew, Springer-Verlag GmbH: 201-221.
100. Oren T, Ehud A, Levy O. Arthroplasty for Fractures. In: Huri G, Paschos NK, editors. The shoulder; 2017. (ISBN No. ISBN 978-3-319-51979-1)
101. Perez, E. (2017) Fractures of the Shoulder, Arm, and Forearm, in: Campbell's Operative Orthopaedics, Chapter 57, 2927-3016
102. Rommens, P. M. and R. Küchle (2017). Intramedulläre Nagelung proximaler Humerusfrakturen. in: Schulter. L.-J. Lehmann and M. Loew, Springer-Verlag GmbH: 187-201.
103. Saghieh, S.S. and N.S. Madi, Management of Upper Limb Fractures, in Reconstructing the War Injured Patient, G. Abu-Sittah, J. Hoballah, and J. Bakhach, Editors. 2017, Springer: Cham. p. 67-77.
