# Supplementary material for: Complications after surgical management of proximal humeral fractures: a systematic review of event terms and definitions
Source: BMC Musculoskelet Disord. 2020 May 26;21:327. doi: 10.1186/s12891-020-03353-8 (PMC7251821; doi:10.1186/s12891-020-03353-8)
Supplement: Supplementary file 3 — Additional file 3. Extracted unfavorable event terms. [file 12891_2020_3353_MOESM3_ESM.pdf]

Supplementary file 3

Article title Complications after surgical management of proximal humeral fractures: a systematic review of event terms and definitions
Journal name BMC Musculoskeletal Disorders
Author names Alispahic N, Brorson S, Bahrs C, Joeris A, Steinitz A, Audigé L
Corresponding author Laurent Audigé, Schulthess Klinik, CH-8008 Zurich, Switzerland
e-mail address laurent.audige@kws.ch

Extracted unfavorable event terms

Event group and specification terms	Language	Extracted event terms	Non-surgical	Plating	Nailing	Arthroplasty	Other surgical	Number of references
**LOCAL EVENTS**								
**1- Implant events**								
**Migration (subsidence, tilt, shift)**								
Wire and Pin								
	en	wire migration	-	-	-	-	X	1
	en	migration of kirschner-wire	-	-	-	-	X	1
	en	migration of k-wires	-	-	-	-	X	2
	en	k-wire migration	-	-	-	-	X	1
	en	pin migration	-	-	-	-	X	4
	en	painful migration of k-wires	-	-	-	-	X	1
	de	pinwanderung	-	-	-	-	X	1
Prosthesis								
	en	varus mobilization of stem	-	-	-	X	-	1
	en	superior migration of humeral prosthesis	-	-	-	X	-	1
	en	superior migration pf the prosthesis	-	-	-	X	-	1
	en	subsidence and malrotation of the stem	-	-	-	X	-	1
	en	mobilization of the glenoid component	-	-	-	X	-	1
	en	superior migration	-	-	-	X	-	1
	en	migration	-	-	-	X	-	1
	en	implant instability	-	-	-	X	-	1
	en	subsidence of the head	-	X	-	-	-	1
	en	mobilization of the implant	-	X	-	-	-	1
	en	rod migration	-	-	X	-	-	1
	en	hardware migration	-	-	-	-	X	1
	de	sekundäre dislokation des implantates	-	-	X	-	-	1

Event group and specification terms	Language	Extracted event terms	Non-surgical	Plating	Nailing	Arthroplasty	Other surgical	Number of references
**Radiolucency around the implant / Implant loosening**								
	en	signs of loosening	-	-	-	X	-	1
	en	pin loosening	-	-	-	-	X	2
	en	osteolysis	-	-	-	X	-	1
	en	loosening/failure of the devices	-	X	-	X	X	1
	en	loosening of an implant	-	X	-	-	-	1
	en	loosening	-	X	-	X	-	3
	en	humeral implant loosening	-	-	-	X	-	1
	en	implant loosening	-	X	X	X	-	7
	en	humeral loosening	-	-	-	X	-	1
	en	secondary head implant loosening	-	X	-	-	-	1
	en	hardware loosening	-	X	-	-	-	1
	en	aseptic loosening	-	-	-	X	-	2
	de	auslockerung	-	-	-	-	X	1
	de	auslockerung implantat	-	-	X	-	-	1
	de	implantatlockerung	-	-	X	-	-	1
	de	plattenlockerung	-	X	-	-	-	1
Prosthesis	en	stem loosening	-	-	-	X	-	1
	en	prosthetic loosening	-	-	-	X	-	2
	en	component loosening	-	-	-	X	-	2
	en	signs of humeral component loosening	-	-	-	X	-	1
	en	aseptic humeral stem loosening	-	-	-	X	-	1
	en	aseptic glenoid loosening	-	-	-	X	-	1
	en	loosening of the glenosphere	-	-	-	X	-	1
	en	loosening of the glenoid component	-	-	-	X	-	1
	en	glenoid component loosening	-	-	-	X	-	2
	de	glenoidale lockerung	-	-	-	X	-	1
**Breakage**								
	en	screw breakage	-	X	-	-	-	2
	en	plate breakage	-	X	-	-	-	5
	en	nail breakage	-	-	X	-	-	1
	en	intraoperative plate breakage	-	X	-	-	-	1
	en	breakage	-	X	-	-	-	2
	en	hardware breakage	-	X	-	-	-	1
	en	hardware fractures	-	X	-	-	-	1
	en	implant fracture	-	X	-	-	-	1

Event group and specification terms	Language	Extracted event terms	Non-surgical	Plating	Nailing	Arthroplasty	Other surgical	Number of references
Failure	en	hardware failure	-	X	-	-	-	6
	en	implant failure	-	X	X	X	X	17
	en	fatigue failure	-	X	-	-	-	1
	en	device failure	-	X	-	-	-	1
	en	failure	-	X	-	-	-	1
	en	plate failure	-	X	-	-	-	1
	en	screw failure	-	-	X	-	-	1
	en	mechanical failure	-	X	-	-	-	3
	en	construct failure	-	X	X	-	-	3
	de	implantatbruch	-	X	-	-	-	1
	de	implantatversagen	-	-	-	-	X	1
	de	osteosyntheseversagen	-	X	-	-	-	1
Disassembly	en	disassembly	-	X	-	-	-	1
**Malpositioning**								
	en	wrong screw positioning	-	X	X	-	-	1
	en	too high nail	-	-	X	-	-	1
	en	prosthesis malpositioning	-	-	-	X	-	1
	en	primary malposition of the implant	-	X	-	-	-	1
	en	poor plate positioning	-	X	-	-	-	1
	en	overstuffing of the joint	-	-	-	X	-	1
	en	inadequate implant position	-	X	-	-	-	1
	en	improper humeral head position	-	-	-	X	-	1
	en	implant malposition	-	X	-	X	-	2
	en	implant malpositioning	-	-	-	X	-	2
	en	implant malpositioning causing revision	-	-	X	-	-	1
	en	implant protrusion	-	-	X	-	-	1
	en	component malposition	-	-	-	X	-	1
	de	offsetfehler bei zu hoher kopfkalotte	-	-	-	X	-	1
	de	fehlerhafte implantationshöhe	-	-	-	X	-	1
	de	plattenfehllage	-	X	-	-	-	1
	de	horizontale offsetfehler	-	-	-	X	-	1
	de	implantatfehllage	-	X	X	-	X	1
	de	implantatüberstand	-	-	X	-	-	1
	de	torsionsfehler	-	-	-	X	-	1
	de	rotationsfehler	-	-	X	-	-	1

Event group and specification terms	Language	Extracted event terms	Non-surgical	Plating	Nailing	Arthroplasty	Other surgical	Number of references
**Screw or bolt backout / loosening**								
	en	windshield-wipering effect	-	X	-	-	-	1
	en	screw loosening	-	X	X	-	-	4
	en	loosening of the screw	-	X	-	-	-	1
	en	loosening of the proximal screw	-	-	X	-	-	1
	en	loose plate and screws	-	X	-	-	-	1
	en	loosening of a single screw	-	X	-	-	-	1
	en	screw migration	-	X	X	-	X	3
	en	proximal screw migration	-	-	X	-	-	1
	en	screw pullout	-	X	-	-	-	1
	en	screw back-out	-	-	X	-	-	2
	en	screw backing out	-	X	-	-	-	3
	en	pull-out failure	-	X	-	-	-	1
	en	proximal interlocking screw backing out	-	-	X	-	-	1
	en	plate pullout	-	X	-	-	-	2
	en	plate pull-out	-	X	-	-	-	1
	en	lateral backing out	-	-	X	-	-	1
	en	backing out of screws	-	-	X	-	-	1
	en	backing out of the proximal screw	-	-	X	-	-	1
	en	backout	-	X	-	-	-	1
	en	diaphyseal screw back-out	-	X	-	-	-	1
	en	epiphyseal screw back-out	-	X	-	-	-	1
	en	screw failure and backout	-	X	-	-	X	1
	de	backing-out der schrauben	-	-	X	-	-	1
	de	schraubenlockerung	-	X	-	-	-	2
**Hardware local irritation**								
	en	hardware intolerance	-	X	-	-	-	1
	en	hardware irritation	-	X	-	-	-	1
**Other**								
	en	metalwork problems	-	X	X	X	X	1
	en	component misalignement	-	-	-	X	-	1
	de	intraartikulärer zementaustritt	-	X	-	-	-	1

Event group and specification terms	Language	Extracted event terms	Non-surgical	Plating	Nailing	Arthroplasty	Other surgical	Number of references
**2- Osteochondral events**								
**Bone formation / resorption (specifications including notching, osteochondral erosions, and bone cyst)**								
	en	humeral bone loss	-	-	-	X	-	1
Notching	en	clinically silent scapular notching	-	-	-	X	-	1
	en	scapular notching	-	-	-	X	-	15
	en	reverse prosthesis: glenoid notching	-	-	-	X	-	1
	en	groove	-	-	-	X	-	1
	en	scapular neck notching	-	-	-	X	-	1
	en	inferior notching	-	-	-	X	-	1
	en	inferior scapular notch	-	-	-	X	-	1
	en	inferior scapular notching	-	-	-	X	-	1
	en	inferiores glenoid-notching	-	-	-	X	-	1
	en	notches	-	-	-	X	-	1
	en	notching	-	-	-	X	-	2
	de	inferiores skapulanotching	-	-	-	X	-	1
Heterotopic bone formation	en	heterotopic bone formation	X	X	X	X	X	1
	en	ectopic ossification	-	-	-	X	-	2
	en	ectopic bone formation	-	-	-	X	-	2
	en	heterotopic ossification	-	X	X	X	X	7
	en	ossification	-	-	-	X	-	1
	de	heterotoper ossifikationen	-	-	-	X	-	1
Glenoid erosion	en	erosion of the glenoid	-	-	-	X	-	1
	en	secondary glenoid destruction	-	X	-	-	-	1
	en	glenoid destruction	-	X	-	-	-	1
	en	articular cartilage injury	-	-	X	-	-	1
**Arthritis**								
	en	degenerative arthritis	X	X	-	-	-	1
	en	destruction of the proximal articular surface	-	-	X	-	-	1
	en	posttraumatic osteoarthritis	-	X	-	-	-	1
	en	posttraumatic arthritis	-	X	-	-	-	1
	en	postraumatic glenoid degeneration	-	-	-	X	-	1
	en	post-traumatic osteoarthritis	-	X	-	-	-	1
	en	post-traumatic shoulder osteoarthritis according to Gerber	-	X	-	-	-	1
	en	post-traumatic arthritis	X	-	-	-	X	1
	en	posttraumatische arthrose	X	X	X	-	X	1
	de	sekundäre glenoidarthrose	-	-	-	X	-	1

Event group and specification terms	Language	Extracted event terms	Non-surgical	Plating	Nailing	Arthroplasty	Other surgical	Number of references
**Fracture around the implant**								
	en	peri-implant fracture	-	X	-	-	-	2
	en	peri-implant fractures	-	-	X	-	-	1
	en	fracture	-	-	-	X	-	1
	en	iatrogenic fracture	-	-	-	X	-	1
	en	iatrogenic fractures	-	-	X	X	-	2
	en	refracture	-	X	X	-	-	3
	en	distal fracture extension	-	X	-	-	-	1
Periprosthetic	en	traumatic periprosthetic fracture	-	-	-	X	-	1
	en	periprosthetic humeral fracture	-	-	-	X	-	1
	en	intra-operative periprosthetic fracture	-	-	-	X	-	1
	en	intraoperativ periprosthetic fractures	-	-	-	X	-	1
	en	periprosthetic fracture	-	-	-	X	-	3
Nail	en	fracture through the entry point of the nail	-	-	X	-	-	1
Plate	en	fractures distal to the plate	-	X	-	-	-	1
Humerus fracture	en	intraoperative fracture of the humerus	-	-	-	X	-	1
	en	diaphyseal fracture of the humerus	-	-	-	X	-	1
	en	humeral shaft fracture	-	-	-	X	-	1
	en	intraoperative diaphyseal fissure	-	-	-	X	-	1
	en	intraoperative diaphyseal fracture	-	-	-	X	-	1
Tuberosity fracture	en	splitting the greater tuberosity	-	-	X	-	-	1
	en	greater tuberosity fractures	-	-	X	-	-	1
	en	iatrogenic tuberosity fracture	-	-	X	-	-	1
	en	secondary lesser tuberosity fracture dislocation	-	X	-	-	-	1
Acromion fracture	en	acromial fracture	-	-	-	X	-	2
	en	intraoperative fracture of acromion	-	-	-	X	-	1
	en	fracture of the acromion	-	-	-	X	-	1
	en	acromial stress fracture	-	-	-	X	-	1
	en	acromion fracture	-	-	-	X	-	4
Scapula spine fracture	en	spine of scapula fracture	-	-	-	X	-	1
	en	intraoperative fracture of spine of scapula	-	-	-	X	-	1
Glenoid fracture	en	intraoperative glenoid fracture	-	-	-	X	-	1
	en	intraoperative fracture of glenoid	-	-	-	X	-	1
	en	fracture of the glenoid	-	-	-	X	-	1
	en	glenoid fracture	-	-	-	X	-	1
	de	glenoidfrakturen	-	-	-	X	-	1

Event group and specification terms	Language	Extracted event terms	Non-surgical	Plating	Nailing	Arthroplasty	Other surgical	Number of references
**Screw / bolt perforation (intra) & Screw / bolt cutout (postop)**								
Cutout	en	slight screw cutout	-	X	-	-	-	1
	en	secondary screw cut-out	-	X	-	-	-	3
	en	cut-out proximal screws after varus collapse	-	X	-	-	-	1
	en	cutout of the screws	-	X	-	-	-	1
	en	cutting out of screws	-	X	-	-	-	1
	en	screw cutout	-	X	X	-	X	16
	en	angular stable screw cut out	-	-	X	-	-	1
	en	primary screw cutout	-	X	-	-	-	3
	en	screw cut-through	-	X	-	-	-	1
	de	schrauben cut-out	-	X	-	-	-	1
	de	cutout der schrauben	-	X	-	-	-	2
Perforation/Penetration	en	secondary screw perforation	-	X	-	-	-	4
	en	secondary intra-articular screw penetration	-	X	-	-	-	1
	en	screw joint penetration	-	X	-	-	-	1
	en	screw penetration	-	X	X	-	-	16
	en	screw penetration of the joint	-	X	-	-	-	1
	en	screw perforation	-	X	X	X	X	14
	en	screw perforation into the joint space	-	X	-	-	-	1
	en	screw perforation of the humeral head	-	X	-	-	-	1
	en	screw perforation to the head	-	X	-	-	-	1
	en	primary perforation of head screw	-	X	-	-	-	1
	en	pin penetration of cartilage	-	X	-	-	-	1
	en	interlocking screw perforation	-	-	X	-	-	1
	en	humeral head perforation	-	X	-	-	-	1
	en	perforation der kalotte	-	X	X	-	-	2
	en	head perforation	-	X	-	-	-	1
	en	glenohumeral screw penetration	-	-	X	-	-	1
	en	articular screw penetration	-	X	X	-	-	1
	en	articular screw perforation	-	X	-	-	-	1
	en	perforation of head screws	-	X	-	-	-	1
	en	perforation of the humeral head	-	X	-	-	-	1
	en	cortical perforation	-	-	-	X	-	1
	en	intraoperative screw perforation	-	X	-	-	-	1
	en	intra-articular screw	-	X	-	-	-	1
	en	intra-articular screw placement	-	X	-	-	-	1

Event group and specification terms	Language	Extracted event terms	Non-surgical	Plating	Nailing	Arthroplasty	Other surgical	Number of references
	en	intra-articular screw penetration	-	X	-	-	-	4
	en	intra-articular penetration of screws	-	X	-	-	-	1
	en	intra-articular migration of screws	-	X	-	-	-	1
	en	joint penetration	-	X	-	-	-	2
	en	penetration of the proximal screw	-	X	-	-	-	1
	en	primary screw perforation	-	X	-	-	-	3
	en	primäre schraubenperforation	-	X	-	-	-	1
	de	schraubenperforation	-	X	-	-	-	2
	de	nagelperforation	-	-	X	-	-	1
	de	perforation von schraubenspitzen ins gelenk	-	-	X	-	-	1
	de	perforation des implantates durch das kalottenfragment	-	-	X	-	-	1
	de	perforation der spiralklinge durch die knorpelfläche der kopfkalotte	-	-	X	-	-	1
	de	Sekundäre Perforation	-	X	-	-	-	1
	de	durchschneiden des implantates durch den kopf	-	-	X	-	-	1
	en	pin perforation	-	-	-	-	X	1
	en	synthesis material in the intra-articular space	-	X	-	-	X	1
Protrusion	en	primary screw protrusion	-	X	X	-	-	1
	en	protrusion of the osteosynthesis material	-	X	-	-	-	1
	en	secondary screw protrusion	-	X	X	-	-	1
	en	screw/nail protrusion	-	-	X	-	-	1
	en	screw protrusion	-	X	X	-	-	3
	en	nail protrusion	-	-	X	-	-	2
	en	blade plate protrusion	-	X	-	-	-	1
**Tuberosity migration / resorption**								
Migration	en	tuberosity fixation failure	-	-	-	X	-	1
	en	tuberosity retraction	-	-	-	X	-	1
	en	tuberosity displacement	-	X	-	X	-	3
	en	tuberosity migration	-	-	-	X	-	2
	en	tuberosity detachment	-	-	-	X	-	4
	en	tubercle migration	-	-	-	X	-	1
	en	superior migration of the great tuberosity	-	-	-	X	-	1
	en	secondary tuberosity displacement	-	-	X	X	X	2
	en	secondary lesser tuberosity displacement	-	X	-	-	-	1
	en	secondary dislocation of the greater tuberosity	-	X	-	-	-	1
	en	secondary displacement of greater tuberosity	-	-	-	X	-	1

Event group and specification terms	Language	Extracted event terms	Non-surgical	Plating	Nailing	Arthroplasty	Other surgical	Number of references
	en	posterior migration of the great tuberosity	-	-	-	X	-	1
	en	migration of the greater tuberosity	-	-	-	X	-	1
	en	migration of tuberosities	-	-	-	X	-	4
	en	migration der tubercula	-	-	-	X	-	1
	en	increasing degree of tuberosity displacement	-	-	-	X	-	1
	en	greater tuberosity migration	-	X	-	-	-	1
	en	cranial displaced greater tubercle	-	-	X	-	-	1
	en	greater tuberosity displacement	-	-	X	-	-	2
	en	greater tuberosity pull off	-	-	-	X	-	1
	en	displaced greater tuberosity	-	X	-	-	-	1
	en	displaced tuberosities	-	-	-	X	-	1
	en	displaced tuberosity after prosthesis	-	-	-	X	-	1
	en	displacement of the greater tuberosity	-	X	-	-	-	2
	en	displacement of tuberosities	-	-	-	X	-	1
	en	displaced lesser tuberosity fragment	-	X	-	-	-	1
	de	sekundäre dislokation der tubercula	-	-	-	X	-	1
	de	sekundäre dislokation des tuberculum minus	-	-	X	-	-	1
Resorption	en	absorption of the lesser tuberosity	-	-	-	X	-	1
	en	tuberosity resorption	-	X	-	X	X	7
	en	tuberosity osteolysis	-	-	-	X	-	1
	en	tuberosity absorption	-	-	-	X	-	1
	en	tuberosities reabsorption	-	-	-	X	-	2
	en	resorption of the greater tuberosity	-	-	X	X	-	2
	en	resorption of tuberosities	-	-	-	X	-	3
	en	greater tuberosity resorption	-	-	-	X	-	2
	en	partial osteolysis of the greater tuberosity	-	-	-	-	X	1
	en	lysis of great tuberosity	-	-	-	X	-	1
	de	sekundäre resorption der tubercula	-	-	-	X	-	1
**Osteonecrosis**								
Necrosis in general	en	partial avascular necrosis	-	X	-	-	-	1
	en	osteonecrosis	X	X	X	X	X	14
	en	necrosis	-	-	X	-	-	1
	en	avascular necrosis	X	X	X	X	X	29
	en	avn	X	X	-	-	-	2
	de	nekrose	-	X	-	-	-	1
	de	avaskuläre nekrose	X	X	X	-	X	1

Event group and specification terms	Language	Extracted event terms	Non-surgical	Plating	Nailing	Arthroplasty	Other surgical	Number of references
Humeral head necrosis	en	humeral neck necrosis	-	X	-	-	-	1
	en	secondary post-operative osteonecrosis of the humeral head	-	-	X	-	-	1
	en	post-traumatic humeral head necrosis according to Cruess	-	X	-	-	-	1
	en	osteonecrosis of humeral head	-	-	X	-	-	1
	en	osteonecrosis of the humeral head	X	X	X	-	X	10
	en	necrosis of the humeral head	X	X	X	-	X	2
	en	necrosis of the head	-	X	X	-	X	1
	en	avascular necrosis of the humeral articular fragment	-	X	-	-	-	1
	en	avascular necrosis of the humeral head	X	X	-	-	-	9
	en	humeral head necrosis	-	X	X	-	X	9
	en	humeral head osteonecrosis	-	X	-	-	-	2
	en	humeral head avascular necrosis	-	X	-	-	-	1
	en	head avascular necrosis	X	X	X	-	X	1
	de	aseptische kopfnekrose	-	-	X	-	-	1
	de	humeruskopfnekrose	-	X	-	-	-	3
Tuberosity necrosis	en	avascular necrosis of the humeral head and the tuberosities	-	X	-	-	-	1
	en	avascular necrosis of the tuberosities	-	X	-	-	-	1
	en	secondary osteonecrosis of the tuberosities	-	-	X	-	-	1
	en	tuberosity necrosis	-	-	-	X	-	1
**Delayed / non-union / malunion**								
Delayed union	en	prolonged delayed union	X	X	X	-	X	1
	en	delayed callus formation	-	X	-	-	-	1
	en	delayed healing	-	X	-	-	X	2
	en	delayed union	X	X	X	X	X	6
	de	verzögerte heilung	-	-	X	-	-	1
Nonunion	en	lack of repair	-	X	-	-	-	1
	en	lack of union	-	-	-	X	-	1
	en	pseudarthrosis	X	X	X	X	X	8
	en	fracture nonunion	X	X	X	-	X	3
	en	aseptic non-union	-	X	-	-	-	1
	en	nonunion of the fracture	-	X	-	-	-	1
	en	bone nonunion	-	X	-	-	-	1
	en	lack of consolidation	-	-	-	X	-	1
	en	nonunion	X	X	X	X	X	56
	en	pseudarthrose	X	X	-	-	-	2
	de	ausbleibende frakturheilung	X	X	X	-	X	1

Event group and specification terms	Language	Extracted event terms	Non-surgical	Plating	Nailing	Arthroplasty	Other surgical	Number of references
Tuberosities	en	repair failure of tuberosities	-	-	-	X	-	1
	en	failure of tuberosity healing	-	-	-	X	-	1
	en	tuberosity nonunion	-	-	-	X	-	6
	en	tubercle nonunion	-	-	-	X	-	1
	en	tuberosities nonunion	-	-	-	X	-	1
	en	greater tuberosity non-union	-	-	-	X	-	2
	en	greater tuberosity nonunion	-	X	X	X	-	4
	en	nonunion of the greater tuberosity	-	-	-	X	-	1
	en	nonunion of the tuberosities	-	-	-	X	-	2
	en	pseudoarthrosis of the lesser tuberosity	-	-	-	-	X	1
	en	tuberosity failure	-	-	-	X	-	1
	en	tuberosity malposition	-	-	-	X	-	1
Malunion	en	varus deformity	-	X	X	-	-	3
	en	varus malalignement	-	-	X	-	-	1
	en	posttraumatic malunion	-	-	-	X	-	1
	en	malunited proximal humerus fractures	-	-	-	X	-	1
	en	malunion with varus deformity	-	X	X	-	-	1
	en	malunion	X	X	X	X	X	37
	en	varus malunion	-	X	X	-	-	7
	en	varus malalignment	-	X	-	-	-	2
	en	varus deformity of the surgical neck	-	-	X	-	-	1
	en	secondary varus angulation	-	X	-	-	-	1
	en	secondary varus deformity	-	X	X	-	-	1
	en	secondary varus malalignment	-	X	-	-	-	1
	en	mal-rotation	-	X	-	-	-	1
	de	varusdeformität	-	X	-	-	-	1
	de	varusfehlstellung	X	X	-	-	-	1
	de	varus des kopffragmentes	-	-	X	-	-	1
Tuberosities	en	mal-union of the tuberosity	-	-	-	X	-	1
	en	tuberosity malalignment	-	-	-	X	-	1
	en	tuberosity malunion	-	-	-	X	-	4
	en	malunion of the greater tuberosity	-	-	-	X	-	1
	en	greater tuberosity malunion	X	X	X	X	X	5
	en	lack of healing of tuberosities	-	-	-	X	-	1
	en	malunion of the tuberosities	X	X	X	X	X	4
	en	arm lengthening greater than 20 mm	-	-	-	X	-	1

Event group and specification terms	Language	Extracted event terms	Non-surgical	Plating	Nailing	Arthroplasty	Other surgical	Number of references
**Mal-reduction / loss of reduction / fracture displacement**								
	en	failure of fixation	-	X	-	X	X	3
	en	failure of fracture fixation	-	X	-	-	-	1
Primary fracture reduction	en	tuberosity malposition	-	-	-	X	-	6
	en	varus malposition	-	X	-	-	-	1
	en	varus malreduction	-	X	-	-	-	1
	en	head varus reduction	-	-	-	-	X	1
	en	high head reduction	-	-	-	-	X	1
	en	malreduction	X	X	-	-	X	7
	en	malpositioning of fracture fragments	-	X	-	-	-	1
	en	malpositioning	-	-	-	X	-	1
	en	malposition of tuberosities	-	-	-	X	-	1
	en	malposition of the tuberosities	-	-	X	-	-	1
	en	tuberosity misalignment	-	-	-	X	-	1
	en	malposition des tuberculum majus	-	-	-	X	-	1
	en	malposition	-	X	-	-	-	1
	en	reduced primary fixation stability	-	-	-	-	X	1
	en	poor humeral head fixation	-	X	-	-	-	1
	en	poor head purchase	-	X	-	-	-	1
	en	fracture distraction	X	-	-	-	-	1
	en	mal-positioning of fracture fragments	-	-	X	-	-	1
	en	mal-alignment of fragments	-	-	X	-	-	1
	en	primarily loss of reduction	-	X	-	-	-	1
	en	intraoperative malreduction	-	-	X	-	-	1
	en	insufficient reduction	-	X	-	-	-	2
	en	inadequate medial support	-	X	-	-	-	1
	en	inadequate fracture reduction	-	X	-	-	-	1
	de	ungenügende reposition	-	X	-	-	-	1
	de	distraktionsfehler	-	-	X	-	-	1
	de	malpositionsfehler der tubercula	-	-	-	X	-	1
	de	malpositionierung trochanter minor	X	X	X	-	X	1
Secondary loss of fracture reduction / displacement	en	humeral head collapse	-	X	X	-	-	2
	en	reduction complication	-	X	-	-	-	1
	en	subchondral collapse	-	X	-	-	-	1
	en	fracture collapse	-	X	-	-	-	1

Event group and specification terms	Language	Extracted event terms	Non-surgical	Plating	Nailing	Arthroplasty	Other surgical	Number of references
	en	medial calcar collapse	-	X	-	-	-	1
	en	collapse into varus position	-	X	-	-	-	1
	en	varus re-collapse	-	X	-	-	-	1
	en	varus fracture collapse	-	X	-	-	-	1
	en	varus collapse	X	X	X	-	-	13
	en	fracture collapse into varus	-	X	-	-	-	1
	en	secondary loss of greater tuberosity reduction	-	X	-	-	-	1
	en	loss of reduction major tubercule	-	-	-	X	-	1
	en	loss of reduction in tuberosities	-	-	X	-	-	1
	en	loss of reduction of the greater tuberosity	-	X	X	-	-	1
	en	loss of reduction of greater tuberosity	-	-	-	X	-	1
	en	varus collapse at the fracture site	-	X	-	-	-	1
	en	varus dislocation	-	X	-	-	-	1
	en	displacement	X	-	X	-	-	2
	en	varus displacement	-	X	-	-	-	2
	en	varus displacement of the head	-	X	-	-	-	1
	en	varus displacement of the humeral head	-	X	-	-	-	1
	en	varus or valgus displacement of the humeral head	-	X	-	-	-	1
	en	displacement of the humeral head	-	-	-	-	X	1
	en	head in varus	-	-	-	-	X	1
	en	secondary displacement (cephalic varus)	-	-	-	-	X	1
	en	varus progression	-	X	-	-	-	1
	en	secondary varus displacement	-	X	-	-	-	2
	en	secondary varus dislocation	-	X	-	-	-	1
	en	secondary loss of reduction	-	X	-	-	-	2
	en	secondary tilt	-	X	-	-	-	1
	en	secondary fracture displacement	-	X	-	-	-	1
	en	secondary failure	-	X	-	-	-	1
	en	secondary displacement	X	X	X	X	X	8
	en	secondary dislocation	-	X	-	-	X	1
	en	reduction loss	-	X	X	-	-	2
	en	redislokation tuberculum majus/minus	-	-	X	-	-	1
	en	redislokation tuberculum majus	X	-	-	-	-	1
	en	recurrence of varus deformity	-	X	-	-	-	1
	en	proximal migration of the humerus	-	-	-	X	-	1
	en	re-displacement of fragments	-	-	-	-	X	1

Event group and specification terms	Language	Extracted event terms	Non-surgical	Plating	Nailing	Arthroplasty	Other surgical	Number of references
	en	loss of the reduction	-	X	-	-	-	1
	en	loss of reduction	-	X	X	X	X	20
	en	loss of reduction of humeral head	-	X	X	-	-	1
	en	loss of proximal fixation	-	-	X	-	-	1
	en	loss of medial support	-	-	X	-	-	1
	en	loss of humeral head reduction	-	X	X	-	-	1
	en	loss of head height	-	X	-	-	-	1
	en	loss of fixation of fracture	-	-	X	-	-	1
	en	loss of fixation	-	X	-	X	X	14
	en	postoperative displacement of the fracture site	-	-	X	-	-	1
	en	partial loss of reduction	-	X	-	-	-	1
	en	clinically irrelevant secondary displacement	-	-	-	-	X	1
	en	fracture displacement	X	X	X	-	X	3
	en	fragment displacement	-	X	-	-	-	1
	en	fixation failure	-	X	-	-	-	7
	en	internal fixation failure	-	X	-	-	-	1
	en	fixation failure from disruption of the buttress of the medial calcar	-	X	-	-	-	1
	en	fixation loss	-	X	-	-	-	1
	de	sekundäre varusabkippung	-	X	-	-	-	1
	de	sekundärer korrekturverlust	-	X	X	-	X	1
	de	fragment sekundärdislokation	-	X	-	-	-	1
	de	sekundäre dislokation	-	X	-	-	-	1
	de	repositionsverlust	-	X	-	-	-	1
	de	repositionsverlust mit und ohne schraubenperforation	-	X	-	-	-	1
Other	en	devastation of the periosteum	-	X	-	-	-	1
	en	cement-related thermal necrosis	-	-	-	X	-	1
	en	periosteal stripping	-	X	-	-	-	1
**3- Shoulder instability**								
	en	instability	-	-	-	X	-	8
	en	insufficient primary stability	-	-	X	-	-	1
	en	joint instability	-	-	-	X	-	1
	en	medial instability	-	X	-	-	-	1
	en	instabilität/luxation	-	-	-	X	-	1
	en	instabilität	-	-	-	X	-	1
Subluxation	en	subluxation	-	-	-	X	-	2

Event group and specification terms	Language	Extracted event terms	Non-surgical	Plating	Nailing	Arthroplasty	Other surgical	Number of references
Dislocation	en	anterior dislocation	-	-	-	X	-	2
	en	prosthetic dislocation	-	-	-	X	-	1
	en	posterior dislocation	-	-	-	X	-	1
	en	persistent joint dislocation	-	X	X	-	X	1
	en	luxation	-	-	-	X	-	3
	en	dislocation	-	-	X	X	-	16
	en	dislocation of prosthesis	-	-	-	X	-	1
	en	dislocation of rsa	-	-	-	X	-	1
	en	dislocation of the humeral head	-	X	-	-	-	1
**4- Shoulder pain (idiopathic)**								
	en	pain	X	X	X	X	X	25
	en	painful hardware	-	X	-	-	-	1
	en	persistent pain	X	X	-	X	-	2
	en	chronic pain	-	-	-	X	-	1
	en	shoulder pain	X	-	X	-	-	3
	en	soreness	-	X	-	-	-	1
	de	schmerzen	-	X	X	-	-	2
**5- Peripheral neurological events**								
	en	neurologic complications	-	X	X	X	-	3
	en	neurologic injury	-	X	X	X	-	3
	en	neurological complications	-	-	-	X	-	1
	en	neurological deficit	-	X	-	-	-	1
	en	neurological injuries	-	-	-	X	-	1
	en	neurological injury	-	-	-	X	-	1
Sensory and/or motor disturbance: Cervical or brachial plexus	en	plexus paralysis	-	-	-	X	-	1
	en	brachial plexopathy	-	-	-	X	-	1
	en	brachial plexus lesion	-	X	-	-	-	1
	en	brachial plexus nerve injury	-	-	-	X	-	1
	en	brachial plexus palsy	-	X	X	X	X	1
Sensory and/or motor disturbance: Branch neuropathy	en	postoperative nerve damage	-	X	-	-	-	1
	en	peripheral nerve injury	-	X	-	X	-	2
	en	palsy	-	-	-	X	-	1
	en	paresthesia	-	X	-	-	-	1

Event group and specification terms	Language	Extracted event terms	Non-surgical	Plating	Nailing	Arthroplasty	Other surgical	Number of references
	en	neurapraxia	-	X	-	-	-	1
	en	neural disorder	-	-	-	X	-	1
	en	nerve damage	-	X	-	-	-	1
	en	nerve injuries	-	X	-	X	-	3
	en	nerve injury	-	X	X	X	X	5
	en	nerve palsy	-	X	-	X	-	2
	en	nerv injury	-	X	-	-	-	1
	en	injury to the neurovascular bundle	-	-	-	-	X	1
	en	transient circumflex nerve palsy	-	X	-	-	-	1
	en	injury to the nerve	-	-	-	X	-	1
	en	iatrogenic neurovascular injury	X	X	-	-	-	1
	en	iatrogenic neurological impairement	-	-	-	X	-	1
Axillary nerve	en	axillary nerve damage	-	X	-	-	-	3
	en	transient palsy of the axillary nerve	-	X	-	-	-	1
	en	persistent palsy of the axillary nerve	-	X	-	-	-	1
	en	neuropraxis of the axillary nerve	-	-	-	X	-	1
	en	axillary nerve injury	-	X	-	X	X	11
	en	injury to the axillary nerve	-	X	X	-	X	2
	en	axillary nerve irritation	-	X	-	-	-	1
	en	axillary nerve lesion	-	X	-	-	-	1
	en	iatrogenic injury of axillary nerve	-	X	-	-	X	2
	en	iatrogenic injury to the axillary nerve	-	X	-	-	-	1
	en	iatrogenic neurotmesis of the axillary nerve	-	X	-	-	X	1
	en	axillary nerve palsy	-	X	X	X	X	2
	en	axillary nerve/vascular injury	-	X	X	-	X	1
	en	decreased axillary nerve sensation	-	X	-	-	-	1
	en	injury to anterior branch of the axillary nerve	-	-	-	-	X	1
	de	direkte schädigung nervus axillaris	-	-	X	-	-	1
	de	verletzung n. axiallaris	-	-	X	-	-	1
	de	neuropraxie des nervus (n.) axillaris	-	-	-	X	-	1
Suprascapular nerve	en	iatrogenic injury of suprascapular nerve	-	X	-	-	X	1
Musculocutaneous nerve	en	injury to the musculocutaneus nerve	-	-	-	-	X	1
Radial nerve	en	injury to the radial nerve	-	-	X	-	X	1
	en	radial nerve injury	-	X	-	X	-	2
	en	nervus radialis läsion	-	-	-	X	-	1
	de	radialisläsion	-	X	-	-	-	1

Event group and specification terms	Language	Extracted event terms	Non-surgical	Plating	Nailing	Arthroplasty	Other surgical	Number of references
Median nerve	en	median nerve injury	-	-	-	X	-	1
Ulnar nerve	en	ulnar nerve injury	-	-	-	X	-	1
Autonomic disturbance: CRPS	en	regional pain syndrom	-	-	-	X	-	1
	en	reflex sympathetic dystrophy	-	-	X	X	-	3
	en	algodystrophy	-	-	X	X	-	3
	en	complex pain regional syndrome	-	-	-	X	-	1
	en	complex regional pain syndrom	-	X	X	X	X	6
	de	komplexes regionales schmerzsyndrom	-	X	-	-	-	1
	de	arthrofibrose und CPRS	-	-	-	X	-	1
**6- Vascular events**								
Hematoma which requires evacuation by needle or surgery	en	hematoma	-	X	X	X	X	15
	en	postoperative hematoma	-	X	-	X	-	3
	en	retropectoral hematoma	-	-	-	X	-	1
	de	nachblutung mit relevanter hämatomentwicklung	-	-	-	X	-	1
Superficial and deep thrombosis at the involved extremity	en	injury to cephalic vein	-	-	-	-	X	1
	en	phlebitis of upper limb	-	-	-	X	-	1
	en	venous thromboembolism	-	X	X	X	X	1
Other	en	axillary artery damage	-	-	-	X	-	1
	en	injury to posterior humeral circumflex artery	-	-	-	-	X	1
	en	posterior humeral circumflex artery injury	-	-	-	-	X	1
	en	postoperative vessel damage	-	X	-	-	-	1
	en	vascular complications	-	X	-	-	-	1
	en	vascular compromise	-	X	-	-	-	1
	en	vascular disorder	-	-	-	X	-	1
	en	vascular injuries	-	X	-	-	-	1
	en	vascular injury	-	-	-	X	-	1
	en	vasculo-nervous lesions	-	-	-	X	-	1
**7- Infections**								
	en	infection	-	X	X	X	X	54
	en	infection (propionibacterium acnes)	-	X	X	X	X	1
	de	infektion	-	-	-	X	-	1

Event group and specification terms	Language	Extracted event terms	Non-surgical	Plating	Nailing	Arthroplasty	Other surgical	Number of references
Surgical Site Infections (SSI): superficial	en	superficial wound infection	-	X	X	-	-	3
	en	superficial surgical site infection	-	-	-	X	-	1
	en	superficial skin infection	-	X	-	-	-	1
	en	superficial infection	-	X	X	X	X	7
	en	incision infection	-	X	-	-	-	1
	en	wound infection	-	X	X	X	-	3
	de	wundinfektion	-	X	-	-	-	1
	de	wundinfekt	-	X	-	-	-	1
	de	wundheilungsstörungen und oberflächliche weichteilinfektionen	-	-	-	X	-	1
Surgical Site Infections (SSI): deep	en	pin tract infection	-	-	-	-	X	4
	en	pin tract-related infection	-	-	-	-	X	1
	en	deep infection	-	X	X	X	X	15
	en	deep wound infection	-	X	-	-	-	1
	en	periprosthetic infection	-	-	-	X	-	1
	en	implant related infection	-	X	-	-	-	1
	en	osteomyelitis	-	X	-	-	X	1
	de	tiefe infektion	-	X	X	-	-	1
	de	pintraktinfektion	-	-	-	-	X	1
Superficial or deep	en	soft tissue infection	-	X	-	-	-	1
	en	postoperative infection	-	-	-	X	-	3
Late hematogenous infections	de	prothesenspätinfekt	-	-	-	X	-	1
**8- Superficial soft tissue events**								
Early events	en	wound healing problem	-	X	-	-	-	1
	en	wound dehiscence	-	-	-	X	-	1
	en	wound healing problems	-	X	-	-	-	1
	en	blister	-	X	-	-	-	1
	en	skin numbness	-	X	-	-	-	1
	en	soft-tissue injury	-	-	X	-	-	1
	en	soft-tissue stripping	-	X	-	-	-	1
Late: hypertrophic scar and keloid	en	scar tissue	-	-	-	-	X	1
	en	scarring	-	X	X	-	-	1
	en	shortening of the soft tissue	-	-	-	-	X	1
	en	soft tissue retraction	-	X	-	-	-	1

Event group and specification terms	Language	Extracted event terms	Non-surgical	Plating	Nailing	Arthroplasty	Other surgical	Number of references
**9- Deep soft tissue events**								
Affecting the subacromial / subcoracoidal space	en	bursitis	-	-	X	-	-	1
	en	periarticular adhesions	-	X	-	-	-	1
	en	reduced subacromial space	-	-	-	X	-	1
Impingement	en	soft tissue impingement	-	X	-	-	-	1
	en	post-operative impingement	-	X	-	-	-	1
	en	plate impingement	-	X	-	-	-	2
	en	impingement	X	X	X	X	X	18
	en	impingement syndrome	-	X	-	-	-	3
	en	decreased abduction with impingement	-	-	-	X	-	1
	en	greater tuberosity impingement	-	X	-	-	-	1
	en	hardware impingement	-	X	-	-	-	1
	en	implant impingement	-	X	-	-	-	1
	en	implant related impingement	-	X	-	-	-	2
	en	mechanical impingement	-	-	-	X	-	1
	en	mechanical complications	-	X	-	-	-	1
	de	mechanisches hindernis bei der schulterbewegung	-	-	X	-	-	1
	de	plattenimpingement	-	X	-	-	-	2
Acromial Impingement	en	acromial impingement	-	X	-	-	-	1
	en	syndrome under-acromial	-	-	X	-	-	1
	en	subacromial dislocation of the greater tuberosity	-	X	-	-	-	1
	en	subacromial impingement syndrom	-	X	-	-	-	1
	en	subacromial impingement	X	X	X	X	-	14
	en	plate impingement with the acromion	-	X	-	-	-	1
Affecting the biceps	en	tenosynovitis of the long head of the biceps	-	X	-	-	-	1
	en	biceps pathology	-	X	X	-	X	1
	en	lesion of the long head of the biceps	-	X	-	-	-	1
	en	secondary retraction and pain (biceps)	-	-	-	X	-	1
	de	verletzung lange bizepssehne intraoperativ	-	-	X	-	-	1
Affecting the capsule (shoulder stiffness, metallosis)	en	arthrofibrosis	-	X	-	-	-	2
	en	unable to elevate the arm above the scapular axis	-	-	X	-	-	1
Adhesive capsulitis	en	frozen shoulder	X	X	-	-	-	4
	en	adhesive capsulitis	X	X	-	-	X	3
	en	capsulitis	X	X	X	X	X	3

Event group and specification terms	Language	Extracted event terms	Non-surgical	Plating	Nailing	Arthroplasty	Other surgical	Number of references
	en	stiffness of shoulder	-	-	-	X	-	1
	en	shoulder stiffness	X	X	X	-	-	6
	en	intractable stiffness	-	-	-	X	-	1
	en	postoperative stiffness	-	X	-	-	X	1
	en	stiffness	X	X	X	X	X	20
	de	steifigkeit	X	X	X	-	-	1
Affecting the rotator cuff	en	violation of supraspinatus on greater tuberosity	-	-	X	-	-	1
	en	secondary rotator cuff damage	-	-	-	X	-	1
	en	secondary rotator cuff insufficiency	-	X	-	-	-	1
	en	secondary cuff failure	-	-	-	X	-	1
	en	secondary cuff rupture	-	-	-	X	-	1
	en	rotator cuff pain	X	-	X	-	-	2
	en	rotator cuff symptoms	-	X	X	X	-	3
	en	rotator cuff tear	X	X	X	-	X	1
	en	rotator cuff tendon tear	-	X	X	-	-	2
	en	rotator cuff damage	-	-	X	X	-	2
	en	rotator cuff defect	-	X	-	-	-	1
	en	rotator cuff dysfunction	X	-	X	X	-	5
	en	rotator cuff injury	X	-	X	-	-	3
	en	rotator cuff irritation	-	-	X	-	-	1
	en	rotator cuff lesion	-	-	X	-	-	2
	en	rotator cuff malfunction	-	-	-	X	-	1
	en	iatrogenic rotator cuff injury	-	-	X	-	-	1
	en	hemiarthroplasty: rotator cuff failure	-	-	-	X	-	1
	en	complete rotator cuff tears subscap	-	X	X	-	-	1
	en	complete rotator cuff tears supraspinatus	-	X	X	-	-	1
	en	concomitant lesions of the tendons of the rotator cuff	-	-	X	-	-	1
	en	cuff related complications	-	-	-	X	-	1
	en	cuff tear	-	-	X	X	-	2
	en	cuff tear rupture	-	-	-	X	-	1
	en	cuff violation	-	-	X	-	-	1
	en	damage to the rotator cuff	-	-	X	-	-	1
	en	defect in the supraspinatus	-	X	-	-	-	1
	en	dysfunction of the rotator cuff	X	-	-	X	-	2
	en	disconnection of rotator cuff tendons from tuberosities	-	X	-	-	-	1
	en	excessive tension of the supraspinatus muscle	-	-	-	X	-	1

Event group and specification terms	Language	Extracted event terms	Non-surgical	Plating	Nailing	Arthroplasty	Other surgical	Number of references
	en	extensive tension of the supraspinatus tendon	-	-	-	X	-	1
	de	direkte irritation der rotatorenmanschette	-	-	X	-	-	1
	de	schädigung des ansatzes der rotatorenmanschette	-	-	X	-	-	1
	de	sekundäre rotatorenmanschettendefekte	-	-	-	X	-	1
Affecting the deltoid	en	weakening of deltoid muscle	-	-	-	X	-	1
	en	deltoid muscle damage	-	X	-	-	-	1
	en	deltoid paresis	-	-	-	X	-	1
Other	en	separation of the muscular flap	-	-	-	X	-	1
	en	seroma	-	-	-	X	-	1
**NON-LOCAL EVENTS**								
**1, Anaphylactic / allergic reaction**								
	en	allergic reaction to metals	-	X	-	-	-	1
**2, Neuro-psychiatric event**								
**3, Cardiovascular event**								
	en	sepsis	-	X	X	X	-	2
	en	septic shock	-	-	-	X	-	1
	en	myocardial infarction	-	-	-	X	-	1
	en	acute myocardial infarction	-	-	-	X	-	1
	en	anemia	X	X	X	X	X	1
	en	blood loss	-	X	X	-	-	1
	en	cardiac arrest	-	-	-	X	-	1
	en	deep venous thrombosis	-	-	-	X	-	1
	en	dvt	X	X	X	X	X	2
	en	lymphatic edema	-	-	-	X	-	1
	en	lymphedema	-	-	-	X	-	1
	en	lymphoedema	-	-	-	X	-	1
	en	neurovascular complications	-	X	-	-	-	1
	en	neurovascular damage	-	X	X	-	X	2
	en	neurovascular injury	-	X	X	-	X	2
	en	cerebrovascular accident	-	-	-	X	-	1
**4, Pulmonary event**								
	en	hydropneumothorax	-	-	-	-	X	1
	en	pneumonia	X	X	X	X	X	3
	en	pulmonary embolism	-	-	-	X	X	1

Event group and specification terms	Language	Extracted event terms	Non-surgical	Plating	Nailing	Arthroplasty	Other surgical	Number of references
	en	pulmonary embolus	-	-	-	X	-	1
	en	respiratory failure	-	-	-	X	-	1
**5, Urinary tract event**								
	en	diabetes mellitus with chronic renal failure	-	-	-	X	-	1
**6, Gastrointestinal event**								
**7, Musculoskeletal system**								
	en	decubitus	-	X	-	-	-	1
	en	fibular graft harvest site leg pain	-	X	-	-	-	1
**Excluded terms (no unfavorable events)**								
Too general term	en	local complications	-	-	-	-	X	1
	en	complication of humeral head replacement	-	X	X	X	X	1
	en	complication of surgical fixation	-	X	X	X	X	1
	en	implant related problems	-	X	X	X	X	1
	en	hardware complications	-	-	X	-	-	1
	en	mechanical problems	-	-	-	-	X	1
	en	perioperative morbidity	-	X	X	-	-	1
Influencing factors	en	compromising the vascularization of bone fragments	-	X	-	-	-	1
	en	jeopardizing the survival of the bone fragments	-	X	-	-	-	1
	en	head diaphysis angle	-	X	-	-	-	1
	en	medial metaphysis reconstruction	-	X	-	-	-	1
	en	postoperative anatomy score=quality of reconstruction of anatomy	-	X	-	-	-	1
	en	greater tuberosity height	-	X	-	-	-	1
	en	lateral metaphyseal comminution	-	-	X	-	-	1
Choice of implant	en	incorrect humeral version	-	X	-	-	-	1
	de	zu lange zementierstrecke	-	-	-	X	-	1
Treatment issues	en	lack of tuberosity fixation to prosthesis	-	-	-	X	-	1
	en	höhere reoperationsrate	-	X	X	-	X	1
	en	need for secondary surgery	-	X	X	X	-	1
	en	secondary removal of the hardware	-	-	-	-	X	1
	en	need for implant removal	-	-	-	-	X	1
	en	hardware removal	-	-	X	-	-	1
	en	metallentfnerung	-	-	-	-	X	1

Excluded terms (no unfavorable events)	Language	Extracted event terms	Non-surgical	Plating	Nailing	Arthroplasty	Other surgical	Number of references
	en	reoperation	-	X	X	-	-	2
	en	revision	-	-	-	X	-	1
	en	non-compliance with rehabilitation	-	-	-	X	-	1
Outcome issues	en	restriction of the glenohumeral joint during arm elevation	-	-	X	-	-	1
	en	restricted abduction	X	X	X	X	X	1
	en	restriction in rom	-	-	X	-	-	1
	en	restriction of internal or external rotation motion and forward flexion	-	-	-	X	-	1
	en	restriction of range of motion	-	-	-	X	-	1
	en	loss of motion	-	-	-	X	-	2
	en	limited rom	-	-	X	-	-	1
	en	decreased range of motion	X	-	-	X	X	2
	en	restriction of strength	-	-	-	X	-	1
	en	loss of function	-	-	-	X	-	1
	en	loss of lifting strength	-	-	-	X	-	1
	en	death	-	X	X	X	X	2
	en	discomfort	-	X	-	-	-	1
