# Supplementary material for: Complications after surgical management of proximal humeral fractures: a systematic review of event terms and definitions
Source: BMC Musculoskelet Disord. 2020 May 26;21:327. doi: 10.1186/s12891-020-03353-8 (PMC7251821; doi:10.1186/s12891-020-03353-8)
Supplement: Supplementary file 4 — Additional file 4. Extracted definitions related to specific event terms. [file 12891_2020_3353_MOESM4_ESM.pdf]

Supplementary file 4

**Article title**	Complications after surgical management of proximal humeral fractures: a systematic review of event terms and definitions
**Journal name**	BMC Musculoskeletal Disorders
**Author names**	Alispahic N, Brorson S, Bahrs C, Joeris A, Steinitz A, Audigé L
**Corresponding author**	Laurent Audigé, Schulthess Klinik, CH-8008 Zurich, Switzerland
**e-mail address**	laurent.audige@kws.ch

Extracted definitions related to specific event terms

Malunion

Authors	Year	Term Used	Definition	Adapted from	Cited by
Sohn et al. [47] †	2014	Malreduction	Head-shaft angle < 120° or > 140°, measured on immediate postoperative radiographs	Brunner et al. 2009 [11]	Sohn et al. 2017 [46]
Bahrs et al.[7]	2015	Malunion	Residual bone deformities at time of follow-up after healing measured on AP and axillary radiographic views (varus / valgus angulation on the AP view [±15°] AND/OR anteversion or retroversion on the axillary view [±15°]) AND/OR tuberosity displacement with reference to the opposite side, with or tuberosity displacement of > 5 mm	Bahrs et al. 2010 [6]	
Okike et al.[36]	2015	Malunion	Healing in varus, valgus or translation alone		
Gracitelli et al. [19]	2016	Varus malunion	Neck-shaft angle ≤ 110°		
Esenyel et al. [14]	2017	Tuberosity malposition (hemiarthroplasty)	Tuberosity position of more than 5 mm above or more than 10mm below the prosthetic head		
Hao et al. [22]	2017	Varus malunion	Neck-shaft angle ≤ 110°		
Villodre-Jimenez et al.[53]	2017	Arm lengthening > 20mm	Difference in the acromion-epicondyle distance in the radiographic checks of the operated arm compared to the contralateral arm	Lädermann et al. 2009 [30]	

† not from the initial literature search

Delayed healing / nonunion

Authors	Year	Term Used	Definition	Adapted from	Cited by
Vannabouathong et al. [51]	2011	Nonunion	Absence of callus uniting the main fracture fragments in 3 of the 4 bone cortices		Gracitelli et al. 2016 [19]
Papakonstantinou et al. [38]	2017	Delayed union / nonunion	Delayed union = Union between 61 and 89 days; it is defined as prolonged after 90 days.<br>Nonunion = When fractures had not united by 90 days	Sheck et al. 1982 [42]	

Secondary fracture displacement

Authors	Year	Term Used	Definition	Adapted from	Cited by
Platzer et al. [40] †	2005	Tuberosity displacement	Displacement >5mm.	Park et al. 1997 [39]	Kancherla et al. 2017 [26]
Ockert et al. [35] †	2010	Secondary varus displacement	Displacement >10° or the CCD angle (centrum collum diaphyseal angle)		Handoll et al. 2015 [21]
Bahrs et al. [7]	2015	Secondary fracture displacement	Secondary fracture displacement at follow-up (defined as > 5° difference of CCD angle or > 5 mm secondary fracture displacement of the greater tuberosity under consideration of the radiographs after fracture stabilization and FU for AP and axillary views and for tuberosity displacement for the AP view).	Brunner et al. 2009 [11]<br>Helwig et al. 2009 [23]<br>Roderer et al. 2011 [41]	
Doursounian et al. [15]	2016	Displacement of the humeral head	More than 10% displacement in the diameter of the humeral head (i.e. 5 mm for 50 mm diameter).		
Doursounian et al. [15]	2016	Displacement of the tuberosities	30° or more change in angle of a bone fragment		
Gonc et al. [18]	2016	Varus progression	Head-shaft angle < 130° postoperatively is considered a sign of varus progression	Acklin et al. 2013 [2]<br>Sohn et al. 2014 [47]	
Gracitelli et al. [19]	2016	Loss of reduction of humeral head	Change of the neck shaft angle ≥ 10°		

Secondary fracture displacement (continued)

Authors	Year	Term Used	Definition	Adapted from	Cited by
Gracitelli et al. [19]	2016	Loss of reduction of the greater tuberosity	Displacement of the greater tuberosity ≥ 0.5 cm		
Hao et al. [22]	2017	Loss of reduction in tuberosities	Displacement of a tuberosity > 5 mm		
Shukla et al. [44]	2017	Varus re-collapse	In patients with an initial varus fracture pattern, neck- shaft angle < 120° as measured on an anteroposterior radiograph with 20° of external rotation.	Agudelo et al. 2007 [4]	
Sohn et al. [46]	2017	Varus collapse	Significantly progressive change of the head-shaft angle with < 120° from the immediate postoperative radiograph to the final follow-up evaluation	Brunner et al. 2009 [11]	
Wolfensperger et al. [55]	2017	Displacement	Migration > 5 mm compared with the postoperative radiographs	Kralinger et al. 2004 [29]	

† not from the initial literature search

Humeral head necrosis

Authors	Year	Term Used	Definition	Adapted from	Cited by
Fjalestad et al. [17]	2012	Avascular necrosis	Classification system:<br>2 = no changes;<br>1 = changes to normal trabecular organization < 50% of humeral head;<br>0 = > 50% or partial collapse		Fjalestad et al. 2014 [16]<br>Handoll et al. 2015 [21]

Implant failure

Authors	Year	Term Used	Definition	Adapted from	Cited by
Haasters et al. [20]	2016	Loss of fixation	Decreased head-shaft angulation of > 10° in the anteroposterior or lateral plane.	Acklin et al. 2012 [1]	
Vijayvargiya et al. [52]	2016	Failure	Backing out of the screw, plate breakage / pull-out, malunion, nonunion or avascular necrosis of humeral head.		

Screw perforation/cut out

Authors	Year	Term Used	Definition	Adapted from	Cited by
Clavert et al. [13]	2010	Screw cutout	Results through high plate position and inadequate screw length	Agudelo et al. 2007 [4]<br>Owsley et al. 2008 [37]	Bohsali et al. 2017 [9]
Lopiz et al. [31]	2014	Protrusion of the osteosynthesis material	Subacromial protrusion with impingement or articular intrusion of the screws		Handoll et al. 2015 [21]
Spross et al. [48]	2014	Secondary screw cut-out	Locked screws do not allow any slip back and the screw tips slowly protrude through the head fragment into the joint, also known as secondary screw cut-out.	Naranja et al. 2000 [33]<br>Jost et al. 2013 [25]	Ayoub et al. 2017 [5]
Konighausen et al. [28]	2015	Secondary perforation (Sekundäre Perforation)	Resintering of separate screws after loss of reduction (Nachsintern einzelner Schrauben bei Repositionsverlust)		
Konighausen et al. [28]	2015	Primary screw perforation (primäre Schraubenperforation)	Intraoperative overlooked iatrogenic screw protrusion (Intraoperativ übersehene Schraubenüberstände, Iatrogen bedingt)		
Aguado et al. [3]	2016	Screw joint penetration	Screw going into the joint		
Brunner et al. [12]	2017	Primary screw perforation (primäre Schraubenperforation)	Already intraoperative apparent screw joint perforation (intraoperativ bereits ersichtliche intraartikuläre Perforation)	Brunner et al. 2009 [11]<br>Südkamp et al. 2009 [49]	

Notching (specific radiological parameter of the reversed prosthesis)

Authors	Year	Term Used	Definition	Adapted from	Cited by
Hernandez et al. [24]	2015	Notching	Notching is the erosion of the scapular neck secondary to its contact with the humeral component of the implant during upper extremity adduction.	Sirveaux et al. 2004 [45]	
Obert et al. [34]	2016	Groove	A groove on the lower side of the neck of the scapula classified according to 1) Sirveaux Classification 2) Nerot Classification	Valenti et al, 2001 [50]<br>Sirveaux et al. 2004 [45]<br>Baulot et al. 2007 [8]<br>Klein et al. 2008 [27]<br>Mattiassich et al. 2013 [32]	

Stiffness

Authors	Year	Term Used	Definition	Adapted from	Cited by
Sohn et al. [47]	2014	Shoulder stiffness	Limitation of active and passive motion compared to the contralateral shoulder in at least two directions (forward flexion < 120° or 50% restriction of external rotation and internal rotation compared to the contralateral side)	Shin et al. 2013 [43]	Sohn et al. 2017 [46]
Bonnevialle et al. [10]	2016	Stiffness	A passive anterior elevation of less than 80°, associated or not with a passive external rotation of the elbow at the side of less than 10° at the final follow-up was considered to be stiffness.		

Nerve lesion

Authors	Year	Term Used	Definition	Adapted from	Cited by
Westphal et al. [54]	2017	Axillary nerve lesion	Electromyography findings were defined as suspect when the distal latency was longer or the amplitude was reduced by more than 50% compared with the healthy shoulder.		

References

1 Acklin Y, Sommer C. Plate fixation of proximal humerus fractures using the minimally invasive anterolateral delta split approach. Oper Orthop Traumatol 2012;24:61-73.

2 Acklin YP, Stoffel K, Sommer C. A prospective analysis of the functional and radiological outcomes of minimally invasive plating in proximal humerus fractures. Injury 2013;44:456-460. doi: 10.1016/j.injury.2012.09.010

3 Aguado HJ, Mingo J, Torres M, Alvarez-Ramos A, Martín-Ferrero MA. Minimally invasive polyaxial locking plate osteosynthesis for 3–4 part proximal humeral fractures: our institutional experience. Injury 2016;47:S22-S28.

4 Agudelo J, Schurmann M, Stahel P, Helwig P, Morgan SJ, Zechel W et al. Analysis of efficacy and failure in proximal humerus fractures treated with locking plates. J Orthop Trauma 2007;21:676-681. doi: 10.1097/BOT.0b013e31815bb09d

5 Ayoub MA, Gad H, El-Tantawy A, Atef A, Seleem OA. Geriatric complex proximal humeral fracture: intraoperative locking plate problems and proposed solutions. Current Orthopaedic Practice 2017;28:70-78. doi: 10.1097/BCO.0000000000000456

6 Bahrs C, Badke A, Rolauffs B, Weise K, Zipplies S, Dietz K et al. Long-term results after non-plate head-preserving fixation of proximal humeral fractures. Int Orthop 2010;34:883-889. doi: 10.1007/s00264-009-0848-4

7 Bahrs C, Kuhle L, Blumenstock G, Stockle U, Rolauffs B, Freude T. Which parameters affect medium- to long-term results after angular stable plate fixation for proximal humeral fractures? J Shoulder Elbow Surg 2015;24:727-732. doi: http://dx.doi.org/10.1016/j.jse.2014.08.009

8 Baulot E, Valenti P, Garaud P, Boileau P, Neyton L, Sirveaux F et al. Résultats des prothèses inversées. Revue de Chirurgie Orthopédique et Réparatrice de l'Appareil Moteur 2007;93:63-92. doi: 10.1016/s0035-1040(07)92712-9

9 Bohsali K, A. Bois, and M. Wirth,. Fractures of the Proximal Humerus, in. In: C.A. Rockwood ea, Editors., editor. Rockwood and Matsen's the shoulder. Elsevier, Inc.; 2017. p. 183-242.

10 Bonnevialle N, Tournier C, Clavert P, Ohl X, Sirveaux F, Saragaglia D et al. Hemiarthroplasty versus reverse shoulder arthroplasty in 4-part displaced fractures of the proximal humerus: Multicenter retrospective study. Orthop Traumatol Surg Res 2016;102:569-573. doi: 10.1016/j.otsr.2016.02.014

11 Brunner F, Sommer C, Bahrs C, Heuwinkel R, Hafner C, Rillmann P et al. Open reduction and internal fixation of proximal humerus fractures using a proximal humeral locked plate: a prospective multicenter analysis. J Orthop Trauma 2009;23:163-172. doi: 10.1097/BOT.0b013e3181920e5b

12 Brunner U. Kopferhaltende Therapie der proximalen Humerusfraktur. In: Habermeyer P, Lichtenberg S, Loew M, Magosch P, Martetschläger F, Tauber M, editors. Schulterchirurgie. München: Elsevier GmbH; 2017. p. 484-534.

13 Clavert P, Adam P, Bevort A, Bonnomet F, Kempf JF. Pitfalls and complications with locking plate for proximal humerus fracture. J Shoulder Elbow Surg 2010;19:489-494. doi: 10.1016/j.jse.2009.09.005

14 CZ. E. Proximal Humerus Fractures. In: Huri G PN, editors., editor. The Shoulder; 2017.

15 Doursounian L, Le Sant A, Mauprivez R, Miquel A, Beauthier-Landauer V. Open reduction and internal fixation of three- and four-part proximal humeral fractures by intra-focal distraction: observational study of twenty five cases. Int Orthop 2016;40:2373-2382. doi: 10.1007/s00264-015-3109-8

16 Fjalestad T, Hole MØ. Displaced proximal humeral fractures: operative versus non-operative treatment—a 2-year extension of a randomized controlled trial. Eur J Orthop Surg Traumatol 2014;24:1067-1073.

17 Fjalestad T, Hole MO, Hovden IA, Blucher J, Stromsoe K. Surgical treatment with an angular stable plate for complex displaced proximal humeral fractures in elderly patients: a randomized controlled trial. J Orthop Trauma 2012;26:98-106. doi: 10.1097/BOT.0b013e31821c2e15

18 Gonc U, Atabek M, Teker K, Tanriover A. Minimally invasive plate osteosynthesis with PHILOS plate for proximal humerus fractures. Acta Orthop Traumatol Turc 2017;51:17-22. doi: 10.1016/j.aott.2016.10.003
19 Gracitelli ME, Malavolta EA, Assuncao JH, Kojima KE, dos Reis PR, Silva JS et al. Locking intramedullary nails compared with locking plates for two- and three-part proximal humeral surgical neck fractures: a randomized controlled trial. J Shoulder Elbow Surg 2016;25:695-703. doi: 10.1016/j.jse.2016.02.003
20 Haasters F, Siebenbürger G, Helfen T, Daferner M, Böcker W, Ockert B. Complications of locked plating for proximal humeral fractures—are we getting any better? J Shoulder Elbow Surg 2016;25:e295-e303.
21 Handoll HH, Brorson S. Interventions for treating proximal humeral fractures in adults. Cochrane Database Syst Rev 2015:CD000434. doi: 10.1002/14651858.CD000434.pub4
22 Hao TD, Huat AWT. Surgical technique and early outcomes of intramedullary nailing of displaced proximal humeral fractures in an Asian population using a contemporary straight nail design. J Orthop Surg (Hong Kong) 2017;25:2309499017713934. doi: https://dx.doi.org/10.1177/2309499017713934
23 Helwig P, Bahrs C, Epple B, Oehm J, Eingartner C, Weise K. Does fixed-angle plate osteosynthesis solve the problems of a fractured proximal humerus? A prospective series of 87 patients. Acta Orthop 2009;80:92-96.
24 Hernández-Elena J, de la Red-Gallego MÁ, Garcés-Zarzalejo C, Pascual-Carra MA, Pérez-Aguilar MD, Rodríguez-López T et al. Treatment of proximal humeral fractures by reverse shoulder arthroplasty: Mid-term evaluation of functional results and Notching. Revista Española de Cirugía Ortopédica y Traumatología (English Edition) 2015;59:413-420. doi: 10.1016/j.recote.2015.09.005
25 Jost B, Spross C, Grehn H, Gerber C. Locking plate fixation of fractures of the proximal humerus: analysis of complications, revision strategies and outcome. J Shoulder Elbow Surg 2013;22:542-549. doi: 10.1016/j.jse.2012.06.008
26 Kancherla VK, Singh A, Anakwenze OA. Management of Acute Proximal Humeral Fractures. J Am Acad Orthop Surg 2017;25:42-52. doi: 10.5435/JAAOS-D-15-00240
27 Klein M, Juschka M, Hinkenjann B, Scherger B, Ostermann PA. Treatment of comminuted fractures of the proximal humerus in elderly patients with the Delta III reverse shoulder prosthesis. J Orthop Trauma 2008;22:698-704. doi: 10.1097/BOT.0b013e31818afe40
28 Königshausen M, Thierbach A, Kübler L, Gessmann J, Godry H, Gothner M et al. Surgical treatment of 3-and 4-part fractures of the humeral head using a polyaxial-locking plate: results and patient satisfaction. Z Orthop Unfall 2015;153:51-58.
29 Kralinger F, Schwaiger R, Wambacher M, Farrell E, Menth-Chiari W, Lajtai G et al. Outcome after primary hemiarthroplasty for fracture of the head of the humerus. A retrospective multicentre study of 167 patients. J Bone Joint Surg Br 2004;86:217-219.
30 Ladermann A, Williams MD, Melis B, Hoffmeyer P, Walch G. Objective evaluation of lengthening in reverse shoulder arthroplasty. J Shoulder Elbow Surg 2009;18:588-595. doi: 10.1016/j.jse.2009.03.012
31 Lopiz Y, Garcia-Coiradas J, Garcia-Fernandez C, Marco F. Proximal humerus nailing: a randomized clinical trial between curvilinear and straight nails. J Shoulder Elbow Surg 2014;23:369-376.
32 Mattiassich G, Marcovici LL, Krifter RM, Ortmaier R, Wegerer P, Kroepfl A. Delta III reverse shoulder arthroplasty in the treatment of complex 3- and 4-part fractures of the proximal humerus: 6 to 42 months of follow up. BMC Musculoskelet Disord 2013;14:231. doi: 10.1186/1471-2474-14-231
33 Naranja RJ, Jr., Iannotti JP. Displaced three- and four-part proximal humerus fractures: evaluation and management. J Am Acad Orthop Surg 2000;8:373-382.

34 Obert L, Saadnia R, Tournier C, Bonnevialle N, Saragaglia D, Sirveaux F et al. Four-part fractures treated with a reversed total shoulder prosthesis: Prospective and retrospective multicenter study. Results and complications. Orthop Traumatol Surg Res 2016;102:279-285. doi: 10.1016/j.otsr.2016.01.019
35 Ockert B, Braunstein V, Kirchhoff C, Korner M, Kirchhoff S, Kehr K et al. Monoaxial versus polyaxial screw insertion in angular stable plate fixation of proximal humeral fractures: radiographic analysis of a prospective randomized study. J Trauma 2010;69:1545-1551. doi: 10.1097/TA.0b013e3181c9b8a7
36 Okike K, Lee OC, Makanji H, Morgan JH, Harris MB, Vrahas MS. Comparison of locked plate fixation and nonoperative management for displaced proximal humerus fractures in elderly patients. Am J Orthop 2015;44:E106-112.
37 Owsley KC, Gorczyca JT. Fracture displacement and screw cutout after open reduction and locked plate fixation of proximal humeral fractures [corrected]. J Bone Joint Surg Am 2008;90:233-240. doi: 10.2106/JBJS.F.01351
38 Papakonstantinou MK, Hart MJ, Farrugia R, Gosling C, Kamali Moaveni A, van Bavel D et al. Prevalence of non-union and delayed union in proximal humeral fractures. ANZ J Surg 2017;87:55-59. doi: https://dx.doi.org/10.1111/ans.13756
39 Park TS, Choi IY, Kim YH, Park MR, Shon JH, Kim SI. A new suggestion for the treatment of minimally displaced fractures of the greater tuberosity of the proximal humerus. Bull Hosp Jt Dis 1997;56:171-176.
40 Platzer P, Kutscha-Lissberg F, Lehr S, Vecsei V, Gaebler C. The influence of displacement on shoulder function in patients with minimally displaced fractures of the greater tuberosity. Injury 2005;36:1185-1189. doi: 10.1016/j.injury.2005.02.018
41 Röderer G, Erhardt J, Kuster M, Vegt P, Bahrs C, Kinzl L et al. Second generation locked plating of proximal humerus fractures—a prospective multicentre observational study. Int Orthop 2011;35:425-432.
42 Scheck M. Surgical treatment of nonunions of the surgical neck of the humerus. Clinical orthopaedics and related research 1982:255-259. doi:
43 Shin S-J, Lee S-Y. Efficacies of corticosteroid injection at different sites of the shoulder for the treatment of adhesive capsulitis. J Shoulder Elbow Surg 2013;22:521-527.
44 Shukla DR, McAnany S, Pean C, Overley S, Lovy A, Parsons BO. The results of tension band rotator cuff suture fixation of locked plating of displaced proximal humerus fractures. Injury 2017;48:474-480. doi: 10.1016/j.injury.2016.12.022
45 Sirveaux F, Leroux J, Roche O, Gosselin O, De Gasperi M, Molé D. Traitement de l'instabilité postérieure de l'épaule par butée iliaque ou acromiale. Revue de Chirurgie Orthopédique et Réparatrice de l'Appareil Moteur 2004;90:411-419. doi: 10.1016/s0035-1040(04)70167-1
46 Sohn HS, Jeon YS, Lee J, Shin SJ. Clinical comparison between open plating and minimally invasive plate osteosynthesis for displaced proximal humeral fractures: A prospective randomized controlled trial. Injury 2017;48:1175-1182. doi: 10.1016/j.injury.2017.03.027
47 Sohn HS, Shin SJ. Minimally invasive plate osteosynthesis for proximal humeral fractures: clinical and radiologic outcomes according to fracture type. J Shoulder Elbow Surg 2014;23:1334-1340. doi: http://dx.doi.org/10.1016/j.jse.2013.12.018
48 Spross C, Jost B, Rahm S, Winklhofer S, Erhardt J, Benninger E. How many radiographs are needed to detect angular stable head screw cut outs of the proximal humerus–a cadaver study. Injury 2014;45:1557-1563.
49 Südkamp N, Bayer J, Hepp P, Voigt C, Oestern H, Kääb M et al. Open reduction and internal fixation of proximal humeral fractures with use of the locking proximal humerus plate: results of a prospective, multicenter, observational study. J Bone Joint Surg Am 2009;91:1320-1328.
50 Valenti PH, Bouttens D, Nerot C. Delta 3 reversed prosthesis for osteoarthritis with massive rotator cuff tear, long term results (>5 years). In: Walch G, Boileau P, Molé D, editors. Shoulder prostheses: two to ten year follow-up. Montpellier: Sauramps Medical; 2001. p. 253-259.

51 Vannabouathong C, Sprague S, Bhandari M. Guidelines for fracture healing assessments in clinical trials. Part I: definitions and endpoint committees. Injury 2011;42:314-316. doi: 10.1016/j.injury.2010.11.048

52 Vijayvargiya M, Pathak A, Gaur S. Outcome Analysis of Locking Plate Fixation in Proximal Humerus Fracture. J Clin Diagn Res 2016;10:RC01-05. doi: 10.7860/JCDR/2016/18122.8281

53 Villodre-Jimenez J, Estrems-Diaz V, Diranzo-Garcia J, Bru-Pomer A. Reverse shoulder arthroplasty in 3 and 4 part proximal humeral fractures in patients aged more than 65 years: Results and complications. Rev Esp Cir Ortop Traumatol 2017;61:43-50. doi: https://dx.doi.org/10.1016/j.recot.2016.09.005

54 Westphal T, Woischnik S, Adolf D, Feistner H, Piatek S. Axillary nerve lesions after open reduction and internal fixation of proximal humeral fractures through an extended lateral deltoid-split approach: electrophysiological findings. J Shoulder Elbow Surg 2017;26:464-471.

55 Wolfensperger F, Gruninger P, Dietrich M, Vollink M, Benninger E, Schlappi M et al. Reverse shoulder arthroplasty for complex fractures of the proximal humerus in elderly patients: impact on the level of independency, early function, and pain medication. J Shoulder Elbow Surg 2017;26:1462-1468. doi: 10.1016/j.jse.2017.01.021
